# Rigorous formulation of space-charge wake function and impedance by solving the three-dimensional Poisson equation

**DOI:** 10.1038/s41598-018-30960-2

**Published:** 2018-08-24

**Authors:** Yoshihiro Shobuda, Yong Ho Chin

**Affiliations:** 1 JAEA, 2-4 Shirakata, Tokaimura, Nakagun, Ibaraki 319-1195 Japan; 2 KEK, High Energy Accelerator Research Organization, 1-1 Oho, Tsukuba, Ibaraki 305-0801 Japan

## Abstract

In typical numerical simulations, the space-charge force is calculated by slicing a beam into many longitudinal segments and by solving the two-dimensional Poisson equation in each segment. This method neglects longitudinal leakage of the space-charge force to nearby segments owing to its longitudinal spread over 1/*γ*. By contrast, the space-charge impedance, which is the Fourier transform of the wake function, is typically calculated directly in the frequency-domain. So long as we follow these approaches, the longitudinal leakage effect of the wake function will remain to be unclear. In the present report, the space-charge wake function is calculated directly in the time domain by solving the three-dimensional Poisson equation for a longitudinally Gaussian beam. We find that the leakage effect is insignificant for a bunch that is considerably longer than the chamber radius so long as the segment length satisfies a certain condition. We present a criterion for how finely a bunch should be sliced so that the two-dimensional slicing approach can provide a good approximation of the three-dimensional exact solution.

## Introduction

In numerical simulations of beam behavior with the space-charge force including the image charge effects on a beam chamber (so-called “indirect space-charge force”), a beam is sliced into many longitudinal segments and the space-charge force is calculated by solving the two-dimensional Poisson equation in each segment and is applied to the particles in that segment. This method may provide a good approximation in the case of a relativistic beam whose electro-magnetic fields are compressed to a pancake-like disc. However, for a non-relativistic beam, it is not obvious how this approximation is applicable because the electro-magnetic fields are spread out longitudinally at an angle of 1/*γ*, where *γ* is the Lorentz-*γ*. This effect is more profound in the indirect space-charge force because the image charge is spread out over 2*a*/*γ*, where *a* is the chamber radius.

The transverse and longitudinal space-charge wake functions for a relativistic beam are typically approximated by the *δ*-function and its derivative, respectively^1^. Their behaviors for a non-relativistic beam have not been studied thoroughly. Specifically, the wake function and its Fourier transform, that is, impedance, of the indirect space-charge force is not well known for non-relativistic beams. To answer these questions, we must calculate the wake functions of space-charge force three-dimensionally and compare them with the corresponding two-dimensional approximations.

In the present study, we derive the transverse wake function and the impedance of space-charge force, including the indirect space-charge force on a chamber wall for a beam with arbitrary energy. We investigate the condition under which the three-dimensional space-charge force can be well approximated using an ensemble of two-dimensional space-charge forces obtained by solving the Poisson equation in each segment. This exercise helps determine how finely the beam must be divided into segments to satisfy the above condition.

At first, we start with a review of the conventional formula of transverse space-charge impedance. Subsequently, we derive the space-charge wake function by solving the three-dimensional Poisson equation for a Gaussian beam. The space-charge impedance is obtained by performing Fourier transformation of the wake function. In this process, we find that the new formula is slightly different from the conventional formula reviewed in the first section. The cause of this discrepancy is identified by deriving the transverse space-charge impedance directly in the frequency domain. We also investigate the characteristics of the transverse space-charge wake function numerically for various chamber radii and bunch lengths with a fixed transverse beam offset. Before concluding this paper, we compare the three-dimensionally obtained exact space-charge wake function with an ensemble of approximate ones obtained by bunch slicing and by solving the two-dimensional Poisson equation in each segment. In this procedure, we will discuss how finely must the bunch be sliced so that the results obtained using the two-dimensional slicing method agree well with the exact solution. Our concluding remarks are given in the final section.

## Conventional Formulas for the Transverse Space-Charge Impedance

The transverse wake function *W*_*T*_(*ξ*) and the impedance *Z*_*T*_(*ω*) are exchangeable concepts. In general, the wake function of an ultra-relativistic beam must satisfy the causality condition. Namely, it must vanish at the front (including the very position) of the source particle. However, the space-charge wake function is an exceptional case, because it must be longitudinally symmetrical around the source particle.

R. Gluckstern^2^ derived the formula for determining the transverse space-charge impedance for the case that a beam with a transverse offset *r*_*b*_ passes through a chamber of radius *a*:

1 $${Z}_{T}(\omega )=-\,\frac{jL{Z}_{0}{I}_{1}(\bar{k}{r}_{b})}{\beta {\gamma }^{2}\pi {r}_{b}^{2}}[{K}_{1}(\bar{k}{r}_{b})-\frac{{K}_{1}(\bar{k}a)}{{I}_{1}(\bar{k}a)}{I}_{1}(\bar{k}{r}_{b})].$$

Here *I*_*n*_(*z*) and *K*_*n*_(*z*) are the modified Bessel functions^3^, *j* is an imaginary unit, *Z*_0_ is the impedance of free space, *L* is total chamber length, *ω* is angular frequency, *k* = *ω*/*cβ*, *c* is velocity of light, $\bar{k}=k/\gamma$, and, *β* and *γ* are Lorentz*-β* and *γ*, respectively. Note that the impedance is purely imaginary.

Accordingly, the space-charge impedance of an ultra-relativistic beam is typically approximated as follows:

2 $${Z}_{T}(\omega )=-\,\frac{jL{Z}_{0}}{2\pi \beta {\gamma }^{2}}(\frac{1}{{r}_{b}^{2}}-\frac{1}{{a}^{2}}).$$

Thus, the corresponding space-charge wake function is described by the *δ*-function, which violates the causality condition because the *δ*-function is an even function. Because the space-charge force spreads out over 1/*γ* symmetrically in the longitudinal direction, the force excited by the source particle can affect the particles in its front.

To understand more clearly whether this *δ*-function description of the wake function is a good approximation, especially for a non-relativistic beam, we should calculate the wake function directly in the time domain, instead of calculating the Fourier transformed impedance from the beginning of its derivation.

In the next section, we calculate the wake function by solving the three-dimensional Poisson equation in a cylindrical chamber for a ring-shaped beam with a Gaussian longitudinal distribution.

## Three-Dimensional Approach for Determining Space-Charge Transverse Wake Function

Let us start by solving the Poisson equation in cylindrical coordinates for an axisymmetric beam in the rest frame $(c\bar{t},\rho ,\theta ,\bar{z})$^4^, in a perfectly conductive cylindrical chamber of radius *a*. The Poisson equation is as follows:

3 $$\frac{1}{\rho }\frac{\partial }{\partial \rho }(\rho \frac{\partial \bar{{\rm{\Phi }}}}{\partial \rho })+\frac{1}{{\rho }^{2}}\frac{{\partial }^{2}\bar{{\rm{\Phi }}}}{\partial {\theta }^{2}}+\frac{{\partial }^{2}\bar{{\rm{\Phi }}}}{\partial {\bar{z}}^{2}}=-\,c{Z}_{0}{\bar{\rho }}_{p}(\rho ,\theta ,\bar{z}),$$

where the shape of the beam distribution is assumed to be

4 $${\bar{\rho }}_{p}(\rho ,\theta ,\bar{z})={i}_{1}\hat{\rho }(\bar{z})\frac{1}{\pi {r}_{b}^{2}}\delta (\rho -{r}_{b})\,\cos \,\theta ,$$

5 $$\hat{\rho }(\bar{z})=\frac{{e}^{-\frac{{\bar{z}}^{2}}{2{\bar{\sigma }}_{z}^{2}}}}{\sqrt{2\pi }{\bar{\sigma }}_{z}},$$

6 $${\bar{\sigma }}_{z}=\gamma {\sigma }_{z},$$

where $\bar{{\rm{\Phi }}}$ is the scalar potential in the rest frame, *i*_1_ = *qr*_*b*_ is the dipole moment, and *σ*_*z*_ is the longitudinal rms beam size in the lab-frame (*ct*, *ρ*, *θ*, *z*).

The scalar potential Φ and the vector potential *A*_*z*_ in the lab-frame are obtained by the following transformations

7 $${\rm{\Phi }}(\rho ,\theta ,z-\beta ct)=\gamma \bar{{\rm{\Phi }}}(\rho ,\theta ,\gamma (z-\beta ct)),$$

8 $${A}_{z}(\rho ,\theta ,z-\beta ct)=\frac{\beta }{c}\gamma \bar{{\rm{\Phi }}}(\rho ,\theta ,\gamma (z-\beta ct)),$$

respectively.

The Green function $G(\overrightarrow{r},\overrightarrow{r}^{\prime} )$, which satisfies the boundary condition: *G* = 0 at *ρ* = *a*, is given by^5,6^

9 $$\begin{array}{rcl}G(\overrightarrow{r},\overrightarrow{r}^{\prime} ) & = & \sum _{m=0}^{\infty }\,\frac{{\varepsilon }_{m}}{2{\pi }^{2}}\,\cos \,m(\theta -\theta ^{\prime} )\\  &  & \times \,\{\begin{array}{ll}{\int }_{0}^{\infty }\,d\lambda [{K}_{m}(\lambda \rho ^{\prime} )-\tfrac{{K}_{m}(\lambda a)}{{I}_{m}(\lambda a)}{I}_{m}(\lambda \rho ^{\prime} )]\,{I}_{m}(\lambda \rho )\,\cos \,\lambda (\bar{z}-\bar{z}^{\prime} ), & {\rm{for}}\,\rho ^{\prime}  > \rho ,\\ {\int }_{0}^{\infty }\,d\lambda [{K}_{m}(\lambda \rho )-\tfrac{{K}_{m}(\lambda a)}{{I}_{m}(\lambda a)}{I}_{m}(\lambda \rho )]\,{I}_{m}(\lambda \rho ^{\prime} )\,\cos \,\lambda (\bar{z}-\bar{z}^{\prime} ), & {\rm{for}}\,\rho ^{\prime}  < \rho ,\end{array}\end{array}$$

where $\overrightarrow{r}=(\rho ,\theta ,\bar{z}),\overrightarrow{r}^{\prime} =(\rho ^{\prime} ,\theta ^{\prime} ,\bar{z}^{\prime} )$, *ε*_*m*_ = 2 − *δ*_*m*0_ and *δ*_*mn*_ is the Kronecker-*δ* (the derivation is given in the appendix). By using the Green function $G(\overrightarrow{r},\overrightarrow{r}^{\prime} )$, the solution $\bar{{\rm{\Phi }}}$ is calculated as

10 $$\begin{array}{rcl}\bar{{\rm{\Phi }}}(\rho ,\theta ,\bar{z}) & = & {\int }_{0}^{\infty }\,d\lambda {\int }_{-\infty }^{\infty }\,d\bar{z}^{\prime} \,{\int }_{\rho }^{a}\,\rho ^{\prime} d\rho ^{\prime} \,{\int }_{0}^{2\pi }\,d\theta ^{\prime} \,\sum _{m=0}^{\infty }\,\frac{c{Z}_{0}{\varepsilon }_{m}}{2{\pi }^{2}}\,\cos \,m(\theta -\theta ^{\prime} )\\  &  & \times \,[{K}_{m}(\lambda \rho ^{\prime} )-\frac{{K}_{m}(\lambda a)}{{I}_{m}(\lambda a)}{I}_{m}(\lambda \rho ^{\prime} )]{I}_{m}(\lambda \rho )\,\cos \,\lambda (\bar{z}-\bar{z}^{\prime} )\\  &  & \times \,{i}_{m}\frac{{e}^{-\frac{\bar{z}{\text{'}}^{2}}{2{\bar{\sigma }}_{z}^{2}}}}{\sqrt{2\pi }{\bar{\sigma }}_{z}}\frac{1}{\pi {r}_{b}^{2}}\delta (\rho ^{\prime} -{r}_{b})\,\cos \,\theta ^{\prime} \\  &  & +\,{\int }_{0}^{\infty }\,d\lambda \,{\int }_{-\infty }^{\infty }\,d\bar{z}^{\prime} \,{\int }_{0}^{\rho }\,\rho ^{\prime} d\rho ^{\prime} \,{\int }_{0}^{2\pi }\,d\theta ^{\prime} \,\sum _{m=0}^{\infty }\,\frac{c{Z}_{0}{\varepsilon }_{m}}{2{\pi }^{2}}\,\cos \,m(\theta -\theta ^{\prime} )\\  &  & \times \,[{K}_{m}(\lambda \rho )-\frac{{K}_{m}(\lambda a)}{{I}_{m}(\lambda a)}{I}_{m}(\lambda \rho )]{I}_{m}(\lambda \rho ^{\prime} )\,\cos \,\lambda (\bar{z}-\bar{z}\text{'})\\  &  & \times \,{i}_{m}\frac{{e}^{-\frac{\bar{z}{\text{'}}^{2}}{2{\bar{\sigma }}_{z}^{2}}}}{\sqrt{2\pi }{\bar{\sigma }}_{z}}\frac{1}{\pi {r}_{b}^{2}}\delta (\rho ^{\prime} -{r}_{b})\,\cos \,\theta ^{\prime} .\end{array}$$

It is simplified as

11 $$\bar{{\rm{\Phi }}}(\rho ,\theta ,\bar{z})=\frac{c{Z}_{0}{i}_{1}}{{\pi }^{2}{r}_{b}}\,\cos \,\theta \,{\int }_{0}^{\infty }\,d\lambda [{K}_{1}(\lambda \rho )-\frac{{K}_{1}(\lambda a)}{{I}_{1}(\lambda a)}{I}_{1}(\lambda \rho )]{I}_{1}(\lambda {r}_{b}){e}^{-\frac{{\lambda }^{2}{\bar{\sigma }}_{z}^{2}}{2}}\,\cos \,\lambda \bar{z},$$

for *ρ* > *r*_*b*_, and

12 $$\bar{{\rm{\Phi }}}(\rho ,\theta ,\bar{z})=\frac{c{Z}_{0}{i}_{1}}{{\pi }^{2}{r}_{b}}\,\cos \,\theta \,{\int }_{0}^{\infty }\,d\lambda [{K}_{1}(\lambda {r}_{b})-\frac{{K}_{1}(\lambda a)}{{I}_{1}(\lambda a)}{I}_{1}(\lambda {r}_{b})]{I}_{1}(\lambda \rho ){e}^{-\frac{{\lambda }^{2}{\bar{\sigma }}_{z}^{2}}{2}}\,\cos \,\lambda \bar{z},$$

for *ρ* < *r*_*b*_, where we use the formula:

13 $$\frac{1}{\sqrt{2\pi }{\bar{\sigma }}_{z}}\,{\int }_{-\infty }^{\infty }\,d\bar{z}^{\prime} {e}^{-\frac{\bar{z}{^{\prime} }^{2}}{2{\bar{\sigma }}_{z}^{2}}}\,\cos \,\lambda (\bar{z}-\bar{z}^{\prime} )={e}^{-\frac{{\lambda }^{2}{\bar{\sigma }}_{z}^{2}}{2}}\,\cos \,\lambda \bar{z}.$$

To obtain the wake function, we need the scalar Φ and the vector *A*_*z*_ potentials in the lab-frame for *ρ* < *r*_*b*_. By substituting Eq. (12) into Eqs (7) and (8), they are calculated as

14 $$\begin{array}{rcl}{\rm{\Phi }}(\rho ,\theta ,z-c\beta t) & = & \frac{\gamma c{Z}_{0}{i}_{1}}{{\pi }^{2}{r}_{b}}\,\cos \,\theta \\  &  & \times \,{\int }_{0}^{\infty }\,d\lambda [{K}_{1}(\lambda {r}_{b})-\frac{{K}_{1}(\lambda a)}{{I}_{1}(\lambda a)}{I}_{1}(\lambda {r}_{b})]\\  &  & \times \,{I}_{1}(\lambda \rho ){e}^{-\frac{{\lambda }^{2}{\gamma }^{2}{\sigma }_{z}^{2}}{2}}\,\cos \,\lambda \gamma (z-c\beta t),\end{array}$$

15 $$\begin{array}{rcl}{A}_{z} & = & \frac{\gamma \beta {Z}_{0}{i}_{1}}{{\pi }^{2}{r}_{b}}\,\cos \,\theta \\  &  & \times \,{\int }_{0}^{\infty }\,d\lambda [{K}_{1}(\lambda {r}_{b})-\frac{{K}_{1}(\lambda a)}{{I}_{1}(\lambda a)}{I}_{1}(\lambda {r}_{b})]\\  &  & \times \,{I}_{1}(\lambda \rho ){e}^{-\frac{{\lambda }^{2}{\gamma }^{2}{\sigma }_{z}^{2}}{2}}\,\cos \,\lambda \gamma (z-c\beta t),\end{array}$$

which leads to the electro-magnetic fields for *ρ* < *r*_*b*_ as

16 $$\begin{array}{rcl}{E}_{\rho } & = & -\frac{\gamma c{Z}_{0}{i}_{1}}{{\pi }^{2}{r}_{b}}\,\cos \,\theta \\  &  & \times \,{\int }_{0}^{\infty }\,d\lambda [{K}_{1}(\lambda {r}_{b})-\frac{{K}_{1}(\lambda a)}{{I}_{1}(\lambda a)}{I}_{1}(\lambda {r}_{b})]\\  &  & \times \,\frac{\partial {I}_{1}(\lambda \rho )}{\partial \rho }{e}^{-\frac{{\lambda }^{2}{\gamma }^{2}{\sigma }_{z}^{2}}{2}}\,\cos \,\lambda \gamma (z-c\beta t),\end{array}$$

17 $$\begin{array}{rcl}{E}_{\theta } & = & \frac{\gamma c{Z}_{0}{i}_{1}}{\rho {\pi }^{2}{r}_{b}}\,\sin \,\theta \\  &  & \times \,{\int }_{0}^{\infty }\,d\lambda [{K}_{1}(\lambda {r}_{b})-\frac{{K}_{1}(\lambda a)}{{I}_{1}(\lambda a)}{I}_{1}(\lambda {r}_{b})]\\  &  & \times \,{I}_{1}(\lambda \rho ){e}^{-\frac{{\lambda }^{2}{\gamma }^{2}{\sigma }_{z}^{2}}{2}}\,\cos \,\lambda \gamma (z-c\beta t),\end{array}$$

18 $$\begin{array}{rcl}{B}_{\rho } & = & -\frac{\gamma \beta {Z}_{0}{i}_{1}}{\rho {\pi }^{2}{r}_{b}}\,\sin \,\theta \\  &  & \times \,{\int }_{0}^{\infty }\,d\lambda [{K}_{1}(\lambda {r}_{b})-\frac{{K}_{1}(\lambda a)}{{I}_{1}(\lambda a)}{I}_{1}(\lambda {r}_{b})]\\  &  & \times \,{I}_{1}(\lambda \rho ){e}^{-\frac{{\lambda }^{2}{\gamma }^{2}{\sigma }_{z}^{2}}{2}}\,\cos \,\lambda \gamma (z-c\beta t),\end{array}$$

19 $$\begin{array}{rcl}{B}_{\theta } & = & -\beta \frac{\gamma {Z}_{0}{i}_{1}}{{\pi }^{2}{r}_{b}}\,\cos \,\theta \\  &  & \times \,{\int }_{0}^{\infty }\,d\lambda [{K}_{1}(\lambda {r}_{b})-\frac{{K}_{1}(\lambda a)}{{I}_{1}(\lambda a)}{I}_{1}(\lambda {r}_{b})]\\  &  & \times \,\frac{\partial {I}_{1}(\lambda \rho )}{\partial \rho }{e}^{-\frac{{\lambda }^{2}{\gamma }^{2}{\sigma }_{z}^{2}}{2}}\,\cos \,\lambda \gamma (z-c\beta t).\end{array}$$

Since the Lorentz force is given by

20 $$\begin{array}{rcl}{F}_{\rho } & = & {E}_{\rho }-c\beta {B}_{\theta }\\  & = & -\frac{c{Z}_{0}{i}_{1}}{\gamma {\pi }^{2}{r}_{b}}\,\cos \,\theta \,{\int }_{0}^{\infty }\,d\lambda [{K}_{1}(\lambda {r}_{b})-\frac{{K}_{1}(\lambda a)}{{I}_{1}(\lambda a)}{I}_{1}(\lambda {r}_{b})]\\  &  & \times \,\frac{\partial {I}_{1}(\lambda \rho )}{\partial \rho }{e}^{-\frac{{\lambda }^{2}{\gamma }^{2}{\sigma }_{z}^{2}}{2}}\,\cos \,\lambda \gamma (z-c\beta t),\end{array}$$

21 $$\begin{array}{rcl}{F}_{\theta } & = & {E}_{\theta }+c\beta {B}_{\rho }\\  & = & \frac{c{Z}_{0}{i}_{1}}{\rho \gamma {\pi }^{2}{r}_{b}}\,\sin \,\theta \,{\int }_{0}^{\infty }\,d\lambda [{K}_{1}(\lambda {r}_{b})-\frac{{K}_{1}(\lambda a)}{{I}_{1}(\lambda a)}{I}_{1}(\lambda {r}_{b})]\\  &  & \times \,{I}_{1}(\lambda \rho ){e}^{-\frac{{\lambda }^{2}{\gamma }^{2}{\sigma }_{z}^{2}}{2}}\,\cos \,\lambda \gamma (z-c\beta t),\end{array}$$

we can define the force ${\tilde{F}}_{\rho }(\xi )$ in the radial direction felt by the witness particle, which is located at a distance *ξ* from the source particle, as

22 $$\begin{array}{rcl}{\tilde{F}}_{\rho }(\xi ) & = & -\frac{c{Z}_{0}{i}_{1}}{\gamma {\pi }^{2}{r}_{b}}\,\cos \,\theta \,{\int }_{0}^{\infty }\,d\lambda [{K}_{1}(\lambda {r}_{b})-\frac{{K}_{1}(\lambda a)}{{I}_{1}(\lambda a)}{I}_{1}(\lambda {r}_{b})]\frac{\partial {I}_{1}(\lambda \rho )}{\partial \rho }{e}^{-\frac{{\lambda }^{2}{\gamma }^{2}{\sigma }_{z}^{2}}{2}}\\  &  & \times \,{\int }_{-\frac{L}{2}}^{\frac{L}{2}}\,ds\,\cos \,\lambda \gamma (s-c\beta \frac{(s+\xi )}{c\beta })\\  & = & -\frac{Lc{Z}_{0}{i}_{1}}{\gamma {\pi }^{2}{r}_{b}}\,\cos \,\theta \,{\int }_{0}^{\infty }\,d\lambda [{K}_{1}(\lambda {r}_{b})-\frac{{K}_{1}(\lambda a)}{{I}_{1}(\lambda a)}{I}_{1}(\lambda {r}_{b})]\\  &  & \times \,\frac{\partial {I}_{1}(\lambda \rho )}{\partial \rho }{e}^{-\frac{{\lambda }^{2}{\gamma }^{2}{\sigma }_{z}^{2}}{2}}\,\cos (\lambda \gamma \xi ).\end{array}$$

Note that the sign of *ξ* can be positive or negative.

Accordingly, the transverse space-charge wake function *W*_*T*_(*ξ*) is expressed as

23 $$\begin{array}{rcl}{W}_{T}(\xi ) & = & -\frac{Lc{Z}_{0}}{2\gamma {\pi }^{2}{r}_{b}}\,{\int }_{0}^{\infty }\,d\lambda [{K}_{1}(\lambda {r}_{b})-\frac{{K}_{1}(\lambda a)}{{I}_{1}(\lambda a)}{I}_{1}(\lambda {r}_{b})]\\  &  & \times \,\lambda {e}^{-\frac{{\lambda }^{2}{\gamma }^{2}{\sigma }_{z}^{2}}{2}}\,\cos (\lambda \gamma \xi ).\end{array}$$

which violates the causality condition, because the wake function *W*_*T*_(*ξ*) is the even function of *ξ*. Hereinafter, we call the first term in Eq. (23)

24 $${W}_{T,direct}(\xi )=-\,\frac{Lc{Z}_{0}}{2\gamma {\pi }^{2}{r}_{b}}\,{\int }_{0}^{\infty }\,d\lambda {K}_{1}(\lambda {r}_{b})\lambda {e}^{-\frac{{\lambda }^{2}{\gamma }^{2}{\sigma }_{z}^{2}}{2}}\,\cos (\lambda \gamma \xi ),$$

the direct space-charge wake function, and the second term

25 $${W}_{T,indirect}(\xi )=\frac{Lc{Z}_{0}}{2\gamma {\pi }^{2}{r}_{b}}\,{\int }_{0}^{\infty }\,d\lambda \frac{{K}_{1}(\lambda a)}{{I}_{1}(\lambda a)}{I}_{1}(\lambda {r}_{b})\lambda {e}^{-\frac{{\lambda }^{2}{\gamma }^{2}{\sigma }_{z}^{2}}{2}}\,\cos (\lambda \gamma \xi ),$$

the indirect space-charge wake function owing to the image charge on the chamber wall at *ρ* = *a*. Only the direct space-charge wake function *W*_*T*,*direct*_(*ξ*) contributes to the total wake function *W*_*T*_(*ξ*) in open space.

Because the transverse impedance *Z*_*T*_(*ω*) is defined as

26 $${Z}_{T}(\omega )=j\,{\int }_{-\infty }^{\infty }\,\frac{d\xi }{c\beta }{e}^{-j\omega \frac{\xi }{c\beta }}{W}_{T}(\xi ),$$

it is finally expressed as

27 $${Z}_{T}(\omega )=-\,j\frac{L{Z}_{0}}{2\beta {\gamma }^{2}\pi {r}_{b}}(\frac{\omega }{c\beta \gamma })\,[{K}_{1}(\frac{\omega }{c\beta \gamma }{r}_{b})-\frac{{K}_{1}(\frac{\omega }{c\beta \gamma }a)}{{I}_{1}(\frac{\omega }{c\beta \gamma }a)}{I}_{1}(\frac{\omega }{c\beta \gamma }{r}_{b})]\,{e}^{-\frac{{\omega }^{2}{\sigma }_{z}^{2}}{2{c}^{2}{\beta }^{2}}},$$

which becomes

28 $${Z}_{T}(\omega )=-\,j\frac{L{Z}_{0}}{2\beta {\gamma }^{2}\pi }(\frac{1}{{r}_{b}^{2}}-\frac{1}{{a}^{2}}){e}^{-\frac{{\omega }^{2}{\sigma }_{z}^{2}}{2{c}^{2}{\beta }^{2}}},$$

for $\gamma \gg 1$. It reproduces Eq. (2) for an infinitesimal beam with *σ*_*z*_ = 0. In this description, the first and the second terms correspond to the direct and the indirect space-charge impedances, respectively.

Surprisingly, Eq. (27) is different from Eq. (1) even for *σ*_*z*_ = 0. To identify the causes, we recalculate the impedance in the frequency domain by following the conventional analysis.

## Derivation of Impedance in the Frequency Domain

The Maxwell equations can be written as wave equations by assuming that electro-magnetic fields have a time dependency of *e*^*jωt*^. They are described as

29 $$({\rm{\Delta }}+{k}^{2}{\beta }^{2})\overrightarrow{E}=jk\beta {Z}_{0}\overrightarrow{j}+\overrightarrow{\nabla }(c{Z}_{0}\bar{\rho }),$$

30 $$({\rm{\Delta }}+{k}^{2}{\beta }^{2})\overrightarrow{H}=-\,\overrightarrow{\nabla }\times \overrightarrow{j},$$

where $\bar{\rho }$ and $\overrightarrow{j}$ are the charge density and current density of the beam, respectively. The charge density is expressed as

31 $$\bar{\rho }=\frac{{i}_{m}}{{r}_{b}^{1+m}}\delta (\rho -{r}_{b}){\delta }_{p}(\theta )\delta (z-\beta ct)=\sum _{m=0}^{\infty }\,\int \,\frac{dk}{2\pi }{i}_{m}{\rho }_{m},$$

32 $${\rho }_{m}=\frac{1}{\pi {r}_{b}^{1+m}(1+{\delta }_{m0})}\delta (\rho -{r}_{b})\,\cos \,m\theta {e}^{-jk(z-\beta ct)},$$

33 $${i}_{m}=q{r}_{b}^{m},$$

where *δ*_*p*_(*θ*) is the periodic *δ*-function.

In the cylindrical coordinates (*ρ*, *θ*, *z*) for an axially symmetric structure, the wave equations of the longitudinal component of the electric and magnetic field contain no transverse field component. They are decoupled. For the longitudinal field, there is a source term $c{Z}_{0}\partial \bar{\rho }/\partial z+jk\beta {Z}_{0}\,{j}_{z}$, while the *z*-component of $\overrightarrow{\nabla }\times \overrightarrow{j}$ vanishes for particles with longitudinal velocity only.

Because the general solutions of the Maxwell equations are obtained by superposition of the solutions for *i*_*m*_*ρ*_*m*_, we choose *i*_1_*ρ*_1_ as the source term for calculating the transverse impedance. Let us define the source field specified with superscript *S* as the solution that satisfies the Maxwell equations with ${\rho }_{1},{\overrightarrow{j}}_{1}=(0,0,c\beta {\rho }_{1})$ and vanishes at *ρ* → ∞. It is given by

34 $${H}_{z}^{S}=0,$$

35 $${E}_{z}^{S}=\{\begin{array}{ll}\frac{jkc{Z}_{0}{I}_{1}(\bar{k}{r}_{b})}{\pi {r}_{b}{\gamma }^{2}}{K}_{1}(\bar{k}\rho )\,\cos \,\theta {e}^{-jkz} & {\rm{for}}\,\rho  > {r}_{b},\\ \frac{jkc{Z}_{0}{K}_{1}(\bar{k}{r}_{b})}{\pi {r}_{b}{\gamma }^{2}}{I}_{1}(\bar{k}\rho )\,\cos \,\theta {e}^{-jkz} & {\rm{for}}\,{r}_{b} > \rho ,\end{array}$$

36 $$-\frac{{Z}_{0}}{\beta }{H}_{\rho }^{S}={E}_{\theta }^{S}=\{\begin{array}{ll}\frac{c{Z}_{0}{I}_{1}(\bar{k}{r}_{b})}{\rho \pi {r}_{b}}{K}_{1}(\bar{k}\rho )\,\sin \,\theta {e}^{-jkz} & {\rm{for}}\,\rho  > {r}_{b},\\ \frac{c{Z}_{0}{K}_{1}(\bar{k}{r}_{b})}{\rho \pi {r}_{b}}{I}_{1}(\bar{k}\rho )\,\sin \,\theta {e}^{-jkz} & {\rm{for}}\,{r}_{b} > \rho ,\end{array}$$

37 $$\frac{\beta }{{Z}_{0}}{E}_{\rho }^{S}={H}_{\theta }^{S}=\{\begin{array}{ll}\frac{\beta kc{I}_{1}(\bar{k}{r}_{b})}{2\pi {r}_{b}\gamma }({K}_{0}(\bar{k}\rho )+{K}_{2}(\bar{k}\rho ))\,\cos \,\theta {e}^{-jkz} & {\rm{for}}\,\rho  > {r}_{b},\\ -\frac{\beta kc{K}_{1}(\bar{k}{r}_{b})}{2\pi {r}_{b}\gamma }({I}_{0}(\bar{k}\rho )+{I}_{2}(\bar{k}\rho ))\,\cos \,\theta {e}^{-jkz} & {\rm{for}}\,{r}_{b} > \rho ,\end{array}$$

for *m* = 1

Thus, the general solutions of the Maxwell equations for the dipole moment are expressed as

38 $${E}_{\rho }={i}_{1}({E}_{\rho }^{S}-\frac{\gamma \beta kA}{j\omega {\varepsilon }_{0}{Z}_{0}}{I}_{1}^{^{\prime} }(\bar{k}\rho )-\frac{{\beta }^{2}{\gamma }^{2}{I}_{1}(\bar{k}\rho )B}{j\omega {\varepsilon }_{0}\rho }\,\cos \,\theta {e}^{-jkz}),$$

39 $${E}_{\theta }={i}_{1}({E}_{\theta }^{S}-\frac{j\beta \gamma {Z}_{0}}{\bar{k}}(\bar{k}B(k){I}_{1}^{^{\prime} }(\bar{k}\rho )+\frac{A(k)}{{Z}_{0}\beta \rho }{I}_{1}(\bar{k}\rho ))\,\sin \,\theta {e}^{-jkz}),$$

40 $${E}_{z}={i}_{1}({E}_{z}^{S}+A(k){I}_{1}(\bar{k}\rho )\,\cos \,\theta {e}^{-jkz}),$$

41 $${H}_{\rho }={i}_{1}({H}_{\rho }^{S}+A[\frac{{I}_{1}(\bar{k}\rho )}{j\omega {\mu }_{0}\rho }-\frac{{\gamma }^{2}}{j\omega {\mu }_{0}\rho }{I}_{1}(\bar{k}\rho )]-\frac{\gamma {Z}_{0}{I}_{1}^{^{\prime} }(\bar{k}\rho )}{j{\mu }_{0}c}B\,\sin \,\theta {e}^{-jkz}),$$

42 $${H}_{\theta }={i}_{1}({H}_{\theta }^{S}+\frac{j\gamma }{\bar{k}}(\frac{B(k){I}_{1}(\bar{k}\rho )}{\rho }+\frac{\beta \bar{k}A(k)}{{Z}_{0}}{I}_{1^{\prime} }(\bar{k}\rho ))\,\cos \,\theta {e}^{-jkz}),$$

43 $${H}_{z}={i}_{1}B(k){I}_{1}(\bar{k}\rho )\,\sin \,\theta {e}^{-jkz},$$

inside the vacuum chamber (*ρ* < *a*), where *A* and *B* are arbitrary coefficients, *ε*_0_ and *μ*_0_ are the dielectric constant and the permeability of vacuum, respectively.

The perfectly conductive boundary condition at the chamber wall *ρ* = *a* is expressed as

44 $$\frac{jkc{Z}_{0}{I}_{1}(\bar{k}{r}_{b})}{\pi {r}_{b}{\gamma }^{2}}{K}_{1}(\bar{k}a)+A(k){I}_{1}(\bar{k}a)=0,$$

45 $$\frac{c{Z}_{0}{I}_{1}(\bar{k}{r}_{b})}{\rho \pi {r}_{b}}{K}_{1}(\bar{k}a)-\frac{j\beta \gamma {Z}_{0}}{\bar{k}}(\bar{k}B(k){I}_{1}^{^{\prime} }(\bar{k}a)+\frac{A(k)}{{Z}_{0}\beta \rho }{I}_{1}(\bar{k}a))=0.$$

Accordingly, the longitudinal electric field and the Lorentz-force are expressed as

46 $${E}_{z}={i}_{1}[\frac{jkc{Z}_{0}{K}_{1}(\bar{k}{r}_{b})}{\pi {r}_{b}{\gamma }^{2}}{I}_{1}(\bar{k}\rho )-\frac{\frac{jkc{Z}_{0}{I}_{1}(\bar{k}{r}_{b})}{\pi {r}_{b}{\gamma }^{2}}{K}_{1}(\bar{k}a)}{{I}_{1}(\bar{k}a)}{I}_{1}(\bar{k}\rho )]\,\cos \,\theta {e}^{-jkz},$$

47 $${F}_{\rho }={E}_{\rho }-c\beta {\mu }_{0}{H}_{\theta }={i}_{1}{\hat{F}}_{\rho }\,\cos \,\theta ,$$

48 $${F}_{\theta }={E}_{\theta }+c\beta {\mu }_{0}{H}_{\rho }={i}_{1}{\hat{F}}_{\theta }\,\sin \,\theta ,$$

where

49 $${\hat{F}}_{\rho }(\rho )=-\,\frac{c{Z}_{0}\bar{k}{I}_{1}^{^{\prime} }(\bar{k}\rho )}{\pi {r}_{b}{\gamma }^{2}}[{K}_{1}(\bar{k}{r}_{b})-\frac{{I}_{1}(\bar{k}{r}_{b})}{{I}_{1}(\bar{k}a)}{K}_{1}(\bar{k}a)]{e}^{-jkz},$$

50 $${\hat{F}}_{\theta }(\rho )=\frac{c{Z}_{0}{I}_{1}(\bar{k}\rho )}{\rho \pi {r}_{b}{\gamma }^{2}}[{K}_{1}(\bar{k}{r}_{b})-\frac{{I}_{1}(\bar{k}{r}_{b})}{{I}_{1}(\bar{k}a)}{K}_{1}(\bar{k}a)]{e}^{-jkz}.$$

Finally, the transverse impedance is expressed as

51 $$\begin{array}{rcl}{Z}_{T}(\omega ) & = & \frac{j}{c\beta }\,{\int }_{-\frac{L}{2}}^{\frac{L}{2}}\,dz{\hat{F}}_{\rho }(\rho =0){e}^{jkz}=-\,\frac{j}{c\beta }\,{\int }_{-\frac{L}{2}}^{\frac{L}{2}}\,dz{\hat{F}}_{\theta }(\rho =0){e}^{jkz}\\  & = & \frac{L\omega {Z}_{0}}{j2\pi {r}_{b}c{\beta }^{2}{\gamma }^{3}}[{K}_{1}(\bar{k}{r}_{b})-\frac{{K}_{1}(\bar{k}a)}{{I}_{1}(\bar{k}a)}{I}_{1}(\bar{k}{r}_{b})],\end{array}$$

which is identical to Eq. (27) with *σ*_*z*_ = 0^7^, where the first term

52 $${Z}_{T,direct}(\omega )=\frac{L\omega {Z}_{0}}{j2\pi {r}_{b}c{\beta }^{2}{\gamma }^{3}}{K}_{1}(\bar{k}{r}_{b}),$$

is the direct space-charge impedance and the second term

53 $${Z}_{T,indirect}(\omega )=-\,\frac{L\omega {Z}_{0}}{j2\pi {r}_{b}c{\beta }^{2}{\gamma }^{3}}\frac{{K}_{1}(\bar{k}a)}{{I}_{1}(\bar{k}a)}{I}_{1}(\bar{k}{r}_{b}),$$

is the indirect space-charge impedance.

Now, let us examine why Eq. (27) with *σ*_*z*_ = 0 (or Eq. (51)) is different from Eq. (1). The reason is that L. Gluckstern approximates the transverse impedance as

54 $$\begin{array}{rcl}{Z}_{T}(\omega ) & = & -\frac{1}{kc\beta {i}_{1}}\,{\int }_{-\infty }^{\infty }\,\frac{\partial {E}_{z}}{\partial x}{e}^{jkz}\\  & \simeq  & -\frac{1}{2dkc\beta {i}_{1}}\,\int \,dz({E}_{z}(x=d,y=0)-{E}_{z}(x=-\,d,y=0)){e}^{jkz}\\  & = & -\frac{L}{k\pi {r}_{b}c\beta {i}_{1}}\,{\int }_{0}^{2\pi }\,d\theta {E}_{z}({r}_{b},\theta ,z)\,\cos \,\theta {e}^{jkz}.\end{array}$$

If we apply Eqs (46) to (54), we can successfully reproduce Eq. (1).

## Numerical Examples

**Figure 1:**
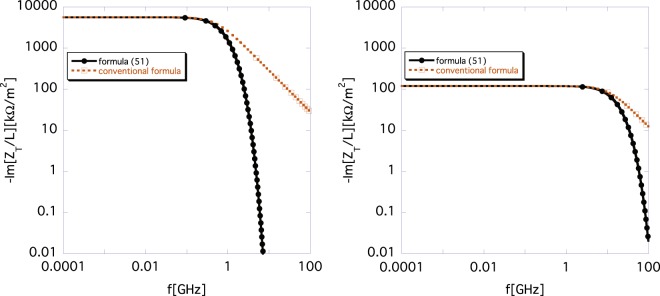
Difference between conventional formula (1) (brown) and new formula (51) (black) for different Lorentz-*β*. The left and the right panels show the imaginary parts of the impedance for *β* = 0.1 and 0.9, respectively.

Now, we compare the conventional formula (1) with the new formula (51) for the parameters used in ref.^8^ (*σ*_*z*_ = 0, *β* = 0.1 or *β* = 0.9; *a* = 40 mm, and *r*_*b*_ = 10mm). The imaginary parts of the impedances *Z*_*T*_(*ω*) for different Lorentz-*β* are shown in Fig. 1. The left and the right panels show the impedances for *β* = 0.1 and 0.9, respectively. The brown and the black lines show the $\Im [{Z}_{T}(\omega )]$ calculated using the conventional formula (1) and the new formula (51), respectively. The space-charge impedances diminish toward the relativistic *γ*, as expected. For both non-relativistic (*β* = 0.1) and relativistic Lorentz-*β* (*β* = 0.9) beams, the space-charge impedances obtained using the new formula (51) are damped more rapidly toward high frequencies than those obtained using Eq. (1). The rapid damping of Eq. (51) toward high frequencies indicates that the wake function obtained by the inverse Fourier transform of the new formula (51) should be more deformed relative to the *δ*-function than that obtained using the conventional formula (1).

Notably, in reference^8^, the numerical simulation results of transverse space-charge impedance were compared with the results obtained using Gluckstern’s formula (1), as in Fig. 5 of ref.^8^, and good agreement was found between them. This is because in that study, the same approximate definition of the transverse impedance (see the definition (54)) as that of Gluckstern was used to derive formula (1). We believe that they can reproduce our results (Fig. 1), if they adopt the exact definition of transverse impedance.

**Figure 2:**
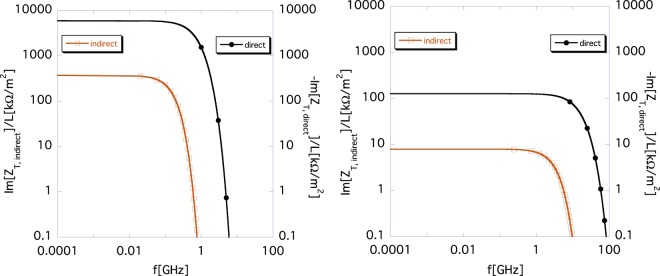
Indirect space-charge impedance *Z*_*T*,*indirect*_(*ω*) (brown) according to Eq. (53) and direct space-charge impedance *Z*_*T*,*direct*_(*ω*) (black) according to Eq. (52) among the new formulas (51) for different Lorentz-*β*. The brown and the black curves are read using the scale markings on the left and the right vertical axes, respectively. The left and the right panels show the results for *β* = 0.1 and 0.9, respectively.

Figure 2 shows the indirect space-charge impedance *Z*_*T*,*indirect*_(*ω*) (brown) calculated using the new formula (53) and the direct one *Z*_*T*,*direct*_(*ω*) (black) calculated using the new formula (52) for different Lorentz-*β*. The left and the right panels show the impedances *Z*_*T*,*indirect*_(*ω*) and *Z*_*T*,*direct*_(*ω*) for *β* = 0.1 and 0.9, respectively. The indirect impedance *Z*_*T*,*indirect*_(*ω*) (brown) is damped more rapidly toward high frequencies than the direct one *Z*_*T*,*direct*_(*ω*) (black), regardless of the Lorentz-*β*, which reflects the fact that the indirect effect originates from the image charge on the chamber wall that spreads out by 2*a*/*γ*. Thus, the direct space-charge impedance *Z*_*T*,*direct*_(*ω*) is dominant in the short range.

**Figure 3:**
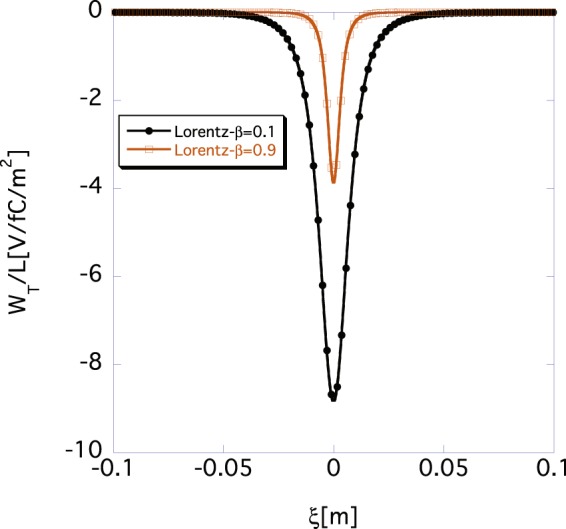
Lorentz-*β* dependence of the wake function *W*_*T*_(*ξ*) according to Eq. (23), where *σ*_*z*_ = 0, *r*_*b*_ = 10 mm, and *a* = 40 mm. The black and brown lines show *W*_*T*_(*ξ*) for *β* = 0.1 and 0.9, respectively.

Next, we investigate the wake function *W*_*T*_(*ξ*) by using Eq. (23) in the time domain for the same set of parameters (*σ*_*z*_ = 0, *β* = 0.1 or *β* = 0.9; *a* = 40 mm, and *r*_*b*_ = 10mm), as in Figs 1 and 2. Figure 3 shows the Lorentz-*β* dependence of the wake function *W*_*T*_(*ξ*). The black and brown lines show the wake functions *W*_*T*_(*ξ*) for *β* = 0.1 and 0.9, respectively. Owing to the relativistic effect, the absolute value of the wake function *W*_*T*_(*ξ*) is smaller for *β* = 0.9 than it is for *β* = 0.1. Again, the transverse wake functions *W*_*T*_(*ξ*) violate the conventional causality condition, because they are even functions of *ξ*.

**Figure 4:**
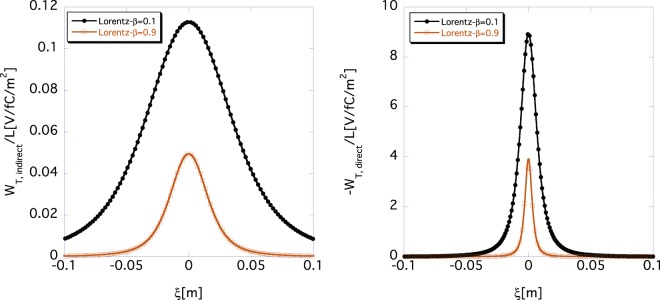
Lorentz-*β* dependence of indirect space-charge wake function *W*_*T*,*indirect*_(*ξ*) according to Eq. (25) (left) and direct one *W*_*T*,*direct*_(*ξ*) according to Eq. (24) (right), where *σ*_*z*_ = 0, *r*_*b*_ = 10 mm, and *a* = 40 mm. The black and brown lines show the wake functions for *β* = 0.1 and 0.9, respectively.

Here, we separate the indirect and the direct contributions of *W*_*T*_(*ξ*). The left and the right panels in Fig. 4 show the indirect wake function *W*_*T*,*indirect*_(*ξ*) obtained using Eq. (25) and the direct one *W*_*T*,*direct*_(*ξ*) obtained using Eq. (24), respectively. The black and the brown lines represent the functions *W*_*T*,*indirect*_(*ξ*) and *W*_*T*,*direct*_(*ξ*) for *β* = 0.1, and for *β* = 0.9, respectively.

Note that the right panel shows the negative values of the direct space-charge wake function *W*_*T*,*direct*_(*ξ*). The indirect wake function *W*_*T*,*indirect*_(*ξ*) and the direct one *W*_*T*,*direct*_(*ξ*) have opposite signs because the former and the latter are generated by the image charge and by a beam, respectively. The indirect space-charge wake function *W*_*T*,*indirect*_(*ξ*) (left) is broader than the direct one (right) because *W*_*T*,*indirect*_(*ξ*) is generated by the image charge on the chamber wall, as expected. Because both *W*_*T*,*indirect*_(*ξ*) and *W*_*T*,*direct*_(*ξ*) become narrower for a larger Lorentz-*γ*, the space-charge wake functions *W*_*T*_(*ξ*) approach the *δ*-functions in an ultra-relativistic beam limit.

**Figure 5:**
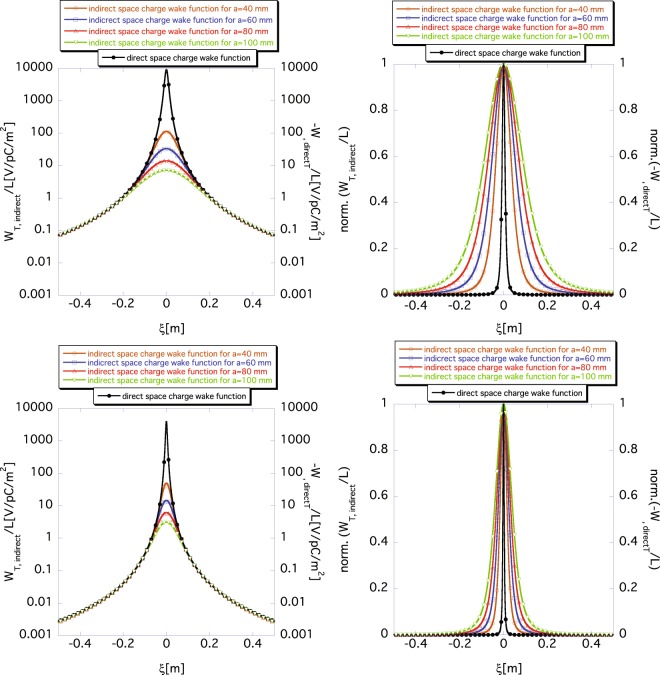
Dependence of indirect space-charge wake function *W*_*T*,*indirect*_(*ξ*) on chamber radius *a* according to Eq. (25), where *σ*_*z*_ = 0 and *r*_*b*_ = 10 mm. The brown, purple, red and green lines (read using the scale markings on the left axis) show the results for *a* = 40 mm, 60 mm, 80 mm, and 100 mm, respectively. The black line (read using the scale marking on the right axis) shows the direct space-charge wake function *W*_*T*,*direct*_(*ξ*) obtained using Eq. (24) as a reference. The upper and lower panels show the functions *W*_*T*,*indirect*_(*ξ*) and *W*_*T*,*direct*_(*ξ*) for *β* = 0.1, and for *β* = 0.9, respectively. The panels on the right show the normalized (to the same amplitude) versions of the panels on the left.

Figure 5 shows the dependence of the indirect space-charge wake function *W*_*T*,*indirect*_(*ξ*) on the chamber radius *a* with fixed beam offset *r*_*b*_ by using Eq. (25). The brown, purple, red and green lines (read using the scale markings on the left axis) represent *W*_*T*,*indirect*_(*ξ*) for *a* = 40 mm, 60 mm, 80 mm, and 100 mm, respectively. The black line (read using the scale markings on the right axis) shows the *W*_*T*,*direct*_(*ξ*) determined using Eq. (24) as a reference. The upper and lower panels show the functions *W*_*T*,*indirect*_(*ξ*) and *W*_*T*,*direct*_(*ξ*) for *β* = 0.1, and for *β* = 0.9, respectively. The panels on the right show the normalized (to the same amplitude) versions of the panels on the left. The shape of the indirect space-charge wake function *W*_*T*,*indirect*_(*ξ*) becomes broader for *β* = 0.1 owing to non-relativistic effects. For both *β* = 0.1 and 0.9, as the chamber radius *a* increases, *W*_*T*,*indirect*_(*ξ*) reduces drastically compared to *W*_*T*,*direct*_(*ξ*). In addition, the shape of the indirect space-charge wake functions *W*_*T*,*indirect*_(*ξ*) becomes broader as the chamber radius increases. Again, this tendency reflects the fact that the indirect space-charge wake function *W*_*T*,*indirect*_(*ξ*) is created by the image charge that spreads out by 2*a*/*γ* on the chamber wall.

**Figure 6:**
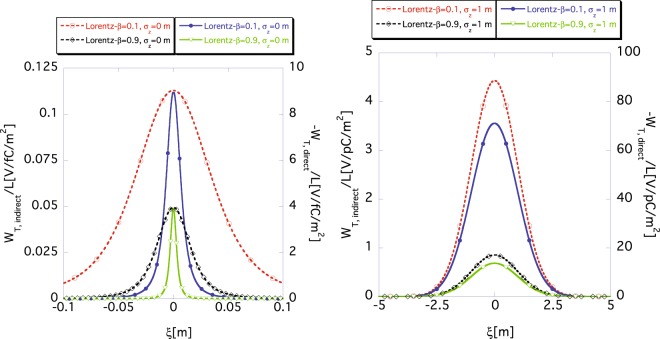
The rms size bunch length *σ*_*z*_ dependence of indirect (dashed) *W*_*T*,*indirect*_(*ξ*) according to Eq. (25) and the direct (solid) wake functions *W*_*T*,*direct*_(*ξ*) according to Eq. (24), where *r*_*b*_ = 10 mm and *a* = 40 mm. The dashed (*W*_*T*,*indirect*_(*ξ*)) and solid (*W*_*T*,*direct*_(*ξ*)) lines are read using the scale markings on the left and the right vertical axes, respectively. The left and the right panels show the wake functions for *σ*_*z*_ = 0 m and 1 m, respectively. The purple and red lines show the wake functions for *β* = 0.1 and the green and black lines for *β* = 0.9.

Finally, the dependences of the indirect wake function *W*_*T*,*indirect*_(*ξ*) and the direct one *W*_*T*,*direct*_(*ξ*) on the rms bunch length *σ*_*z*_ are shown in Fig. 6, which are obtained by using Eqs (24) and (25), respectively, for the case of *r*_*b*_ = 10 mm and *a* = 40 mm, although the wake function *W*_*T*_(*ξ*), or impedance *Z*_*T*_(*ω*), is conventionally defined for infinitesimal *σ*_*z*_ = 0. The left and right panels in Fig. 6 show the functions *W*_*T*,*indirect*_(*ξ*) and *W*_*T*,*direct*_(*ξ*) for *σ*_*z*_ = 0 m, and for *σ*_*z*_ = 1 m, respectively. The dashed (read using the scale marking on the left axis) and the solid (read using the scale marking on the right axis) lines represent *W*_*T*,*indirect*_(*ξ*) and *W*_*T*,*direct*_(*ξ*), respectively. The red dashed and purple solid lines show the wake functions for *β* = 0.1 and the black dashed and green solid lines for *β* = 0.9, respectively. Notice that the scales of the vertical axes are different in the left and the right panels. Figure 6 clearly shows that the wake function *W*_*T*_(*ξ*) is more deformed from the *δ*-function for longer *σ*_*z*_, as expected.

In typical numerical simulations, the space-charge force is obtained by longitudinally slicing a beam into several segments and by solving the two-dimensional Poisson equation. Because we now have the three-dimensionally obtained exact space-charge wake function *W*_*T*_(*ξ*), we can examine the accuracy of this approximation and identify the conditions under which the approximation is adequately accurate. In the next section, we discuss this issue by solving the two- and the three-dimensional Poisson equations, and by comparing the results.

## Comparison of Transverse Space-Charge Wake Functions by Conventional Scheme and Rigorous One

In conventional numerical calculations of space-charge force, the beam is sliced longitudinally into several segments. In each segment, the space-charge force is calculated approximately by solving the two-dimensional Poisson equation, assuming that particles are distributed uniformly in the longitudinal direction in each segment. Let us follow this scheme and approximate the transverse space-charge wake functions theoretically. By comparing the approximated functions with the rigorous ones presented in the section entitled ‘Three-Dimensional Approach for Determining Space-Charge Transverse Wake Function’, we can find out how finely we must slice a beam to obtain a good approximation.

Here, we introduce the two-dimensional Poisson equation in the rest frame $(c\bar{t},\rho ,\theta ,\bar{z})$, which is given by

55 $$\frac{1}{\rho }\frac{\partial }{\partial \rho }(\rho \frac{\partial \bar{{\rm{\Phi }}}(\rho ,\theta ,{\bar{z}}_{i})}{\partial \rho })+\frac{1}{{\rho }^{2}}\frac{{\partial }^{2}\bar{{\rm{\Phi }}}(\rho ,\theta ,{\bar{z}}_{i})}{\partial {\theta }^{2}}=-\,c{Z}_{0}{\bar{\rho }}_{p}(\rho ,\theta ,{\bar{z}}_{i}),\,{\rm{for}}\,{\bar{z}}_{i}-{\rm{\Delta }}\bar{z} < \bar{z} < {\bar{z}}_{i}+{\rm{\Delta }}\bar{z},$$

where the beam distribution in the *i*-th segment within the mesh size $2{\rm{\Delta }}\bar{z}$ is given by

56 $${\bar{\rho }}_{p}(\rho ,\theta ,{\bar{z}}_{i})={i}_{1}\hat{\rho }({\bar{z}}_{i})\frac{1}{\pi {r}_{b}^{2}}\delta (\rho -{r}_{b})\,\cos \,\theta ,$$

57 $$\hat{\rho }({\bar{z}}_{i})=\frac{1}{2{\rm{\Delta }}\bar{z}}\,{\int }_{{\bar{z}}_{i}-{\rm{\Delta }}\bar{z}}^{{\bar{z}}_{i}+{\rm{\Delta }}\bar{z}}\,d\bar{z}\frac{{e}^{-\frac{{\bar{z}}^{2}}{2{\bar{\sigma }}_{z}^{2}}}}{\sqrt{2\pi }{\bar{\sigma }}_{z}}.$$

The scalar potential Φ and the vector potential *A*_*z*_ in the lab-frame are given by

58 $${\rm{\Phi }}(\rho ,\theta ,{z}_{i},t)=\gamma {\bar{{\rm{\Phi }}}}_{i}(\rho ,\theta ,\gamma ({z}_{i}-c\beta t)),$$

59 $${A}_{z}(\rho ,\theta ,{z}_{i},t)=\frac{\beta }{c}\gamma {\bar{{\rm{\Phi }}}}_{i}(\rho ,\theta ,\gamma ({z}_{i}-c\beta t)),$$

for *z*_*i*_ − Δ*z* < *z* < *z*_*i*_ + Δ*z*, respectively, where we use ${\rm{\Delta }}\bar{z}=\gamma {\rm{\Delta }}z$.

The Green function ${G}_{two}(\overrightarrow{r},\overrightarrow{r}^{\prime} )$ for two-dimensional space, which satisfies the boundary condition: *G*_*two*_ = 0 at *ρ* = *a*, is given by (the derivation is given in the appendix)^5,6^

60 $${G}_{two}(\overrightarrow{r},\overrightarrow{r}^{\prime} )=\{\begin{array}{ll}-\frac{1}{2\pi }\,\mathrm{log}\,\frac{\rho ^{\prime} }{a}+\frac{1}{2\pi }\,\sum _{m=1}^{\infty }\,\frac{\cos \,m(\theta -\theta ^{\prime} )}{m}{\rho }^{m}(\frac{1}{{\rho ^{\prime} }^{m}}-\frac{{\rho ^{\prime} }^{m}}{{a}^{2m}}), & {\rm{for}}\,\rho ^{\prime}  > \rho ,\\ -\frac{1}{2\pi }\,\mathrm{log}\,\frac{\rho }{a}+\frac{1}{2\pi }\,\sum _{m=1}^{\infty }\,\frac{\cos \,m(\theta -\theta ^{\prime} )}{m}{\rho ^{\prime} }^{m}(\frac{1}{{\rho }^{m}}-\frac{{\rho }^{m}}{{a}^{2m}}), & {\rm{for}}\,\rho ^{\prime}  < \rho .\end{array}$$

By using the Green function ${G}_{two}(\overrightarrow{r},\overrightarrow{r}^{\prime} )$, the scalar potential $\bar{{\rm{\Phi }}}$ in the rest frame is calculated as

61 $$\begin{array}{rcl}\bar{{\rm{\Phi }}}(\rho ,\theta ,\bar{z}) & = & \frac{c{Z}_{0}}{2{\rm{\Delta }}\bar{z}}\,{\int }_{{\bar{z}}_{i}-{\rm{\Delta }}\bar{z}}^{{\bar{z}}_{i}+{\rm{\Delta }}\bar{z}}\,d\bar{z}^{\prime} \,{\int }_{\rho }^{a}\,\rho ^{\prime} d\rho ^{\prime} \,{\int }_{0}^{2\pi }\,d\theta ^{\prime} \\  &  & \times \,[-\frac{1}{2\pi }\,\mathrm{log}\,\frac{\rho ^{\prime} }{a}+\frac{1}{2\pi }\,\sum _{m=1}^{\infty }\,\frac{\cos \,m(\theta -\theta ^{\prime} )}{m}{\rho }^{m}(\frac{1}{{\rho ^{\prime} }^{m}}-\frac{{\rho ^{\prime} }^{m}}{{a}^{2m}})]\\  &  & \times \,{i}_{1}\frac{{e}^{-\frac{\bar{z}{\text{'}}^{2}}{2{\bar{\sigma }}_{z}^{2}}}}{\sqrt{2\pi }{\bar{\sigma }}_{z}}\frac{1}{\pi {r}_{b}^{2}}\delta (\rho ^{\prime} -{r}_{b})\,\cos \,\theta ^{\prime} \\  &  & +\,\frac{c{Z}_{0}}{2{\rm{\Delta }}\bar{z}}\,{\int }_{{\bar{z}}_{i}-{\rm{\Delta }}\bar{z}}^{{\bar{z}}_{i}+{\rm{\Delta }}\bar{z}}\,d\bar{z}^{\prime} \,{\int }_{0}^{\rho }\,\rho ^{\prime} d\rho ^{\prime} \,{\int }_{0}^{2\pi }\,d\theta ^{\prime} \\  &  & \times \,[-\frac{1}{2\pi }\,\mathrm{log}\,\frac{\rho }{a}+\frac{1}{2\pi }\,\sum _{m=1}^{\infty }\,\frac{\cos \,m(\theta -\theta ^{\prime} )}{m}{\rho ^{\prime} }^{m}(\frac{1}{{\rho }^{m}}-\frac{{\rho }^{m}}{{a}^{2m}})]\\  &  & \times \,{i}_{1}\frac{{e}^{-\frac{\bar{z}{\text{'}}^{2}}{2{\bar{\sigma }}_{z}^{2}}}}{\sqrt{2\pi }{\bar{\sigma }}_{z}}\frac{1}{\pi {r}_{b}^{2}}\delta (\rho ^{\prime} -{r}_{b})\,\cos \,\theta ^{\prime} .\end{array}$$

It is simplified as

62 $$\bar{{\rm{\Phi }}}(\rho ,\theta ,\bar{z})=\frac{c{Z}_{0}}{4\pi {\rm{\Delta }}\bar{z}}(\frac{1}{\rho }-\frac{\rho }{{a}^{2}})\,{i}_{1}\,{\int }_{{\bar{z}}_{i}-{\rm{\Delta }}\bar{z}}^{{\bar{z}}_{i}+{\rm{\Delta }}\bar{z}}d\bar{z}^{\prime} \frac{{e}^{-\frac{\bar{z}{\text{'}}^{2}}{2{\bar{\sigma }}_{z}^{2}}}}{\sqrt{2\pi }{\bar{\sigma }}_{z}}\,\cos \,\theta ,$$

for *ρ* > *r*_*b*_, and

63 $$\bar{{\rm{\Phi }}}(\rho ,\theta ,\bar{z})=\frac{c{Z}_{0}\rho }{4\pi {\rm{\Delta }}\bar{z}{r}_{b}}(\frac{1}{{r}_{b}}-\frac{{r}_{b}}{{a}^{2}})\,{i}_{1}\,{\int }_{{\bar{z}}_{i}-{\rm{\Delta }}\bar{z}}^{{\bar{z}}_{i}+{\rm{\Delta }}\bar{z}}\,d\bar{z}^{\prime} \frac{{e}^{-\frac{\bar{z}{\text{'}}^{2}}{2{\bar{\sigma }}_{z}^{2}}}}{\sqrt{2\pi }{\bar{\sigma }}_{z}}\,\cos \,\theta ,$$

for *ρ* < *r*_*b*_.

To obtain the wake function, we need the scalar Φ and the vector *A*_*z*_ potentials in the lab-frame for *ρ* < *r*_*b*_. They are calculated as

64 $${\rm{\Phi }}(\rho ,\theta ,{z}_{i}-c\beta t)=\frac{c{Z}_{0}\rho }{4\pi {r}_{b}\gamma {\rm{\Delta }}z}(\frac{1}{{r}_{b}}-\frac{{r}_{b}}{{a}^{2}})\,{i}_{1}\,{\int }_{\gamma ({z}_{i}-c\beta t)-\gamma {\rm{\Delta }}z}^{\gamma ({z}_{i}-c\beta t)+\gamma {\rm{\Delta }}z}\,d\bar{z}^{\prime} \frac{{e}^{-\frac{\bar{z}{\text{'}}^{2}}{2{\gamma }^{2}{\sigma }_{z}^{2}}}}{\sqrt{2\pi }{\sigma }_{z}}\,\cos \,\theta ,$$

65 $${A}_{z}(\rho ,\theta ,{z}_{i}-c\beta t)=\frac{c{Z}_{0}\beta \rho }{4c\pi {r}_{b}\gamma {\rm{\Delta }}z}(\frac{1}{{r}_{b}}-\frac{{r}_{b}}{{a}^{2}})\,{i}_{1}\,{\int }_{\gamma ({z}_{i}-c\beta t)-\gamma {\rm{\Delta }}z}^{\gamma ({z}_{i}-c\beta t)+\gamma {\rm{\Delta }}z}\,d\bar{z}^{\prime} \frac{{e}^{-\frac{\bar{z}{\text{'}}^{2}}{2{\gamma }^{2}{\sigma }_{z}^{2}}}}{\sqrt{2\pi }{\sigma }_{z}}\,\cos \,\theta ,$$

by using Eqs (58), (59) and (63), which provide the electro-magnetic fields for *ρ* < *r*_*b*_ as

66 $${E}_{\rho }=-\,\frac{c{Z}_{0}}{4\pi {r}_{b}\gamma {\rm{\Delta }}z}(\frac{1}{{r}_{b}}-\frac{{r}_{b}}{{a}^{2}})\,{i}_{1}\,{\int }_{\gamma ({z}_{i}-c\beta t)-\gamma {\rm{\Delta }}z}^{\gamma ({z}_{i}-c\beta t)+\gamma {\rm{\Delta }}z}\,d\bar{z}^{\prime} \frac{{e}^{-\frac{\bar{z}{\text{'}}^{2}}{2{\gamma }^{2}{\sigma }_{z}^{2}}}}{\sqrt{2\pi }{\sigma }_{z}}\,\cos \,\theta ,$$

67 $${E}_{\theta }=\frac{c{Z}_{0}}{4\pi {r}_{b}\gamma {\rm{\Delta }}z}(\frac{1}{{r}_{b}}-\frac{{r}_{b}}{{a}^{2}})\,{i}_{1}\,{\int }_{\gamma ({z}_{i}-c\beta t)-\gamma {\rm{\Delta }}z}^{\gamma ({z}_{i}-c\beta t)+\gamma {\rm{\Delta }}z}\,d\bar{z}^{\prime} \frac{{e}^{-\frac{\bar{z}{\text{'}}^{2}}{2{\gamma }^{2}{\sigma }_{z}^{2}}}}{\sqrt{2\pi }{\sigma }_{z}}\,\sin \,\theta ,$$

68 $${B}_{\rho }=-\,\frac{c{Z}_{0}\beta }{4c\pi {r}_{b}\gamma {\rm{\Delta }}z}(\frac{1}{{r}_{b}}-\frac{{r}_{b}}{{a}^{2}})\,{i}_{1}\,{\int }_{\gamma ({z}_{i}-c\beta t)-\gamma {\rm{\Delta }}z}^{\gamma ({z}_{i}-c\beta t)+\gamma {\rm{\Delta }}z}\,d\bar{z}^{\prime} \frac{{e}^{-\frac{\bar{z}{\text{'}}^{2}}{2{\gamma }^{2}{\sigma }_{z}^{2}}}}{\sqrt{2\pi }{\sigma }_{z}}\,\sin \,\theta ,$$

69 $${B}_{\theta }=-\,\frac{c{Z}_{0}\beta }{4c\pi {r}_{b}\gamma {\rm{\Delta }}z}(\frac{1}{{r}_{b}}-\frac{{r}_{b}}{{a}^{2}})\,{i}_{1}\,{\int }_{\gamma ({z}_{i}-c\beta t)-\gamma {\rm{\Delta }}z}^{\gamma ({z}_{i}-c\beta t)+\gamma {\rm{\Delta }}z}\,d\bar{z}^{\prime} \frac{{e}^{-\frac{\bar{z}{\text{'}}^{2}}{2{\gamma }^{2}{\sigma }_{z}^{2}}}}{\sqrt{2\pi }{\sigma }_{z}}\,\cos \,\theta .$$

Because the Lorentz force is given by

70 $${F}_{\rho }={E}_{\rho }-c\beta {B}_{\theta }=-\,\frac{c{Z}_{0}}{4\pi {r}_{b}{\gamma }^{3}{\rm{\Delta }}z}(\frac{1}{{r}_{b}}-\frac{{r}_{b}}{{a}^{2}})\,{i}_{1}\,{\int }_{\gamma ({z}_{i}-c\beta t)-\gamma {\rm{\Delta }}z}^{\gamma ({z}_{i}-c\beta t)+\gamma {\rm{\Delta }}z}\,d\bar{z}^{\prime} \frac{{e}^{-\frac{\bar{z}{^{\prime} }^{2}}{2{\gamma }^{2}{\sigma }_{z}^{2}}}}{\sqrt{2\pi }{\sigma }_{z}}\,\cos \,\theta ,$$

71 $${F}_{\theta }={E}_{\theta }+c\beta {B}_{\rho }=\frac{c{Z}_{0}}{4\pi {r}_{b}{\gamma }^{3}{\rm{\Delta }}z}(\frac{1}{{r}_{b}}-\frac{{r}_{b}}{{a}^{2}})\,{i}_{1}\,{\int }_{\gamma ({z}_{i}-c\beta t)-\gamma {\rm{\Delta }}z}^{\gamma ({z}_{i}-c\beta t)+\gamma {\rm{\Delta }}z}\,d\bar{z}^{\prime} \,\frac{{e}^{-\frac{\bar{z}{^{\prime} }^{2}}{2{\gamma }^{2}{\sigma }_{z}^{2}}}}{\sqrt{2\pi }{\sigma }_{z}}\,\sin \,\theta ,$$

for *z*_*i*_ − Δ*z* < *z* < *z*_*i*_ + Δ*z*, the force ${\tilde{F}}_{\rho }(\xi )$ in the radial direction felt by the witness particle, which is at a distance *ξ* from the source particle, is calculated as

72 $${\tilde{F}}_{\rho }(\xi )=-\,\frac{c{Z}_{0}}{4\pi {r}_{b}{\gamma }^{3}{\rm{\Delta }}z}(\frac{1}{{r}_{b}}-\frac{{r}_{b}}{{a}^{2}})\,{i}_{1}\,\sum _{i=1}^{N}\,{\int }_{{z}_{i}-{\rm{\Delta }}z}^{{z}_{i}+{\rm{\Delta }}z}\,ds\,{\int }_{\gamma ({z}_{i}-s-\xi )-\gamma {\rm{\Delta }}z}^{\gamma ({z}_{i}-s-\xi )+\gamma {\rm{\Delta }}z}\,d\bar{z}^{\prime} \frac{{e}^{-\frac{\bar{z}{^{\prime} }^{2}}{2{\gamma }^{2}{\sigma }_{z}^{2}}}}{\sqrt{2\pi }{\sigma }_{z}}\,\cos \,\theta ,$$

where *N* is the number of segments. Finally, the space-charge wake function *W*_*T*_(*ξ*) is summarized as

73 $$\begin{array}{rcl}{W}_{T}(\xi ) & = & -\frac{c{Z}_{0}}{4\pi {r}_{b}{\gamma }^{3}{\rm{\Delta }}z}(\frac{1}{{r}_{b}}-\frac{{r}_{b}}{{a}^{2}})\,\sum _{i=1}^{N}\,{\int }_{{z}_{i}-{\rm{\Delta }}z}^{{z}_{i}+{\rm{\Delta }}z}\,ds\,{\int }_{\gamma ({z}_{i}-s-\xi )-\gamma {\rm{\Delta }}z}^{\gamma ({z}_{i}-s-\xi )+\gamma {\rm{\Delta }}z}\,d\bar{z}^{\prime} \frac{{e}^{-\frac{\bar{z}{^{\prime} }^{2}}{2{\gamma }^{2}{\sigma }_{z}^{2}}}}{\sqrt{2\pi }{\sigma }_{z}}\\  & = & -\frac{c{Z}_{0}}{8\pi {r}_{b}{\gamma }^{2}{\rm{\Delta }}z}(\frac{1}{{r}_{b}}-\frac{{r}_{b}}{{a}^{2}})\,\sum _{i=1}^{N}\,{\int }_{{z}_{i}-{\rm{\Delta }}z}^{{z}_{i}+{\rm{\Delta }}z}\,ds({\rm{Erf}}\,[\frac{{z}_{i}-s-\xi +{\rm{\Delta }}z}{\sqrt{2}{\sigma }_{z}}]\\  &  & -\,{\rm{Erf}}\,[\frac{{z}_{i}-s-\xi -{\rm{\Delta }}z}{\sqrt{2}{\sigma }_{z}}])\\  & = & {W}_{T,direct}(\xi )+{W}_{T,indirect}(\xi ),\end{array}$$

where

74 $$\begin{array}{rcl}{W}_{T,direct}(\xi ) & = & -\frac{c{Z}_{0}L}{16\pi {\gamma }^{2}{\rm{\Delta }}{z}^{2}{r}_{b}^{2}}\{(-{e}^{-\frac{{(\xi -2{\rm{\Delta }}z)}^{2}}{2{\sigma }_{z}^{2}}}+2{e}^{-\frac{{\xi }^{2}}{2{\sigma }_{z}^{2}}}-{e}^{-\frac{{(\xi +2{\rm{\Delta }}z)}^{2}}{2{\sigma }_{z}^{2}}})\sqrt{\frac{2}{\pi }}{\sigma }_{z}\\  &  & -\,2\xi \,{\rm{Erf}}\,[-\frac{\xi }{\sqrt{2}{\sigma }_{z}}]+(\xi -2{\rm{\Delta }}z)\,{\rm{Erf}}\,[-\frac{(\xi -2{\rm{\Delta }}z)}{\sqrt{2}{\sigma }_{z}}]\\  &  & +\,(\xi +2{\rm{\Delta }}z){\rm{Erf}}\,[-\frac{(\xi +2{\rm{\Delta }}z)}{\sqrt{2}{\sigma }_{z}}]\},\end{array}$$

75 $$\begin{array}{rcl}{W}_{T,indirect}(\xi ) & = & -\frac{c{Z}_{0}L}{16\pi {\gamma }^{2}{\rm{\Delta }}{z}^{2}{a}^{2}}\{(-{e}^{-\frac{{(\xi -2{\rm{\Delta }}z)}^{2}}{2{\sigma }_{z}^{2}}}+2{e}^{-\frac{{\xi }^{2}}{2{\sigma }_{z}^{2}}}-{e}^{-\frac{{(\xi +2{\rm{\Delta }}z)}^{2}}{2{\sigma }_{z}^{2}}})\sqrt{\frac{2}{\pi }}{\sigma }_{z}\\  &  & -\,2\xi \,{\rm{Erf}}\,[-\frac{\xi }{\sqrt{2}{\sigma }_{z}}]+(\xi -2{\rm{\Delta }}z)\,{\rm{Erf}}\,[-\frac{(\xi -2{\rm{\Delta }}z)}{\sqrt{2}{\sigma }_{z}}]\\  &  & +\,(\xi +2{\rm{\Delta }}z){\rm{Erf}}\,[-\frac{(\xi +2{\rm{\Delta }}z)}{\sqrt{2}{\sigma }_{z}}]\},\end{array}$$

and Erf[z] is the error function^9^. Again, the wake function *W*_*T*_(*ξ*) is the even function of *ξ*, as in the three dimensional calculation.

At first, we take the limit of Δ*z* to be zero, that is, a beam is sliced into infinite segments. In this limit, Eq. (73) becomes

76 $${W}_{T}(\xi )={W}_{T,direct}(\xi )+{W}_{T,indirect}(\xi ),$$

where

77 $${W}_{T,direct}(\xi )=-\,\frac{c{e}^{-\frac{{\xi }^{2}}{2{\sigma }_{z}^{2}}}L{Z}_{0}}{2\sqrt{2\pi }\pi {r}_{b}^{2}{\gamma }^{2}{\sigma }_{z}},$$

and

78 $${W}_{T,indirect}(\xi )=\frac{c{e}^{-\frac{{\xi }^{2}}{2{\sigma }_{z}^{2}}}L{Z}_{0}}{2\sqrt{2\pi }{a}^{2}\pi {\gamma }^{2}{\sigma }_{z}}.$$

Subsequently, by taking the limit of *σ*_*z*_ as zero, Eq. (76) reproduces

79 $${W}_{T}(\xi )=-\,\frac{cL({a}^{2}-{r}_{b}^{2}){Z}_{0}}{2{\gamma }^{2}{a}^{2}\pi {r}_{b}^{2}}\delta (\xi ),$$

which is the conventional wake function for an ultra-relativistic beam. For a relativistic beam, the longitudinal spread of the space-charge force is assumed to be small from the beginning. In this sense, the reproduction of the conventional formula is consistent with the assumption of neglection of the longitudinal field leakage effect to nearby segments (by 1/*γ* spread of the space-charge force) in this two-dimensional approach.

The transverse impedance *Z*_*T*_(*ω*) can be calculated using Eq. (26). By substituting Eq. (73) into Eq. (26), we obtain

80 $$\begin{array}{rcl}{Z}_{T}(\omega ) & = & j\frac{{Z}_{0}L}{16\pi {r}_{b}\beta {\gamma }^{2}{\rm{\Delta }}{z}^{2}}(\frac{1}{{r}_{b}}-\frac{{r}_{b}}{{a}^{2}})\,{\int }_{-\infty }^{\infty }\,d\xi {e}^{-j\omega \frac{\xi }{c\beta }}\\  &  & \times \,\{(-{e}^{-\frac{{(\xi -2{\rm{\Delta }}z)}^{2}}{2{\sigma }_{z}^{2}}}+2{e}^{-\frac{{\xi }^{2}}{2{\sigma }_{z}^{2}}}-{e}^{-\frac{{(\xi +2{\rm{\Delta }}z)}^{2}}{2{\sigma }_{z}^{2}}})\sqrt{\frac{2}{\pi }}{\sigma }_{z}\\  &  & -\,2\xi \,{\rm{Erf}}\,[-\frac{\xi }{\sqrt{2}{\sigma }_{z}}]+(\xi -2{\rm{\Delta }}z){\rm{Erf}}\,[-\,\frac{(\xi -2{\rm{\Delta }}z)}{\sqrt{2}{\sigma }_{z}}]\\  &  & +\,(\xi +2{\rm{\Delta }}z){\rm{Erf}}\,[-\frac{(\xi +2{\rm{\Delta }}z)}{\sqrt{2}{\sigma }_{z}}]\}\\  & = & -j\frac{{Z}_{0}L{e}^{-\frac{{\omega }^{2}{\sigma }_{z}^{2}}{2{c}^{2}{\beta }^{2}}}\,{\sin }^{2}\,\frac{\omega {\rm{\Delta }}z}{c\beta }}{2\pi \beta {\gamma }^{2}{\rm{\Delta }}{z}^{2}}(\frac{1}{{r}_{b}^{2}}-\frac{1}{{a}^{2}})\frac{{c}^{2}{\beta }^{2}}{{\omega }^{2}},\end{array}$$

which reproduces Eq. (28) for infinitesimal Δ*z*. For *σ*_*z*_ = 0, this becomes the conventional formula (2) of the space-charge impedance for an ultra-relativistic beam, as expected.

**Figure 7:**
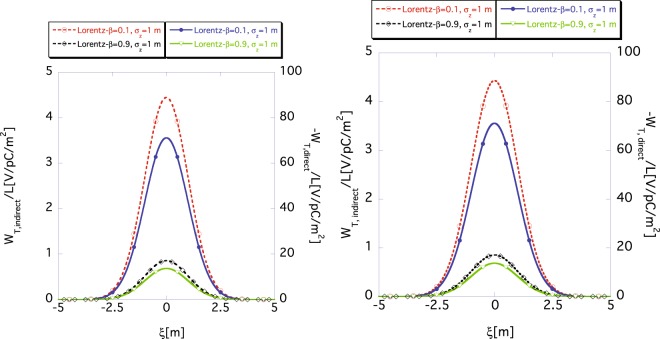
Approximate (left) result obtained using Eqs (77) and (78) and exact (right) one obtained using Eqs (24) and (25) of indirect (dashed) *W*_*T*,*indirect*_(*ξ*) and direct (solid) *W*_*T*,*direct*_(*ξ*) wake functions for *σ*_*z*_ = 1 m, where *r*_*b*_ = 10 mm and *a* = 40 mm. The dashed (*W*_*T*,*indirect*_(*ξ*)) and the solid (*W*_*T*,*direct*_(*ξ*)) lines are read using the scale markings on the left and the right vertical axes, respectively. The purple and red lines show the wake functions for *β* = 0.1 m and the green and black lines for *β* = 0.9 m, respectively.

Here, for a finite bunch length *σ*_*z*_, we investigate how the approximate wake function for infinitesimal Δ*z* can reproduce the exact wake function. The left panel in Fig. 7 shows the approximate indirect wake function *W*_*T*,*indirect*_(*ξ*) (dashed) and the direct one *W*_*T*,*direct*_(*ξ*) (solid) calculated using Eqs (77) and (78), respectively, for *σ*_*z*_ = 1 m. The right panel in Fig. 7 shows the corresponding exact wake functions calculated using Eqs (24) and (25) for the same bunch length. The purple and red lines show the wake functions for *β* = 0.1 m and the green and black lines for *β* = 0.9 m, respectively. The approximate wake functions (left) well reproduce the exact ones (right), which are identical to the ones in the right panel of Fig. 6. Good agreement is obtained in the relativistic and the non-relativistic cases.

**Figure 8:**
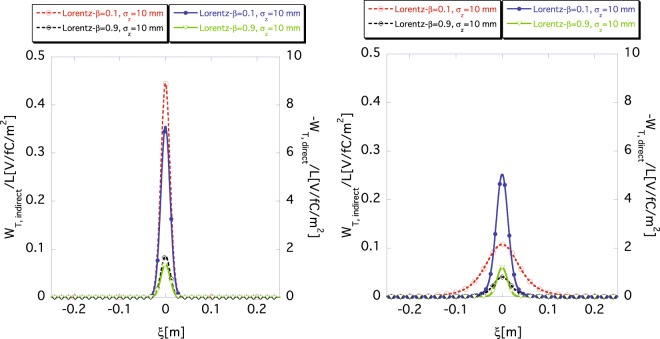
Approximate (left) result obtained using Eqs (77) and (78) and the exact (right) results obtained using Eqs (24) and (25) of the indirect wake function *W*_*T*,*indirect*_(*ξ*) and the direct wake function *W*_*T*,*direct*_(*ξ*) for *σ*_*z*_ = 10 mm, where *r*_*b*_ = 10 mm and *a* = 40 mm. The indirect wake function *W*_*T*,*indirect*_(*ξ*) (dashed) and the direct wake function (solid) *W*_*T*,*direct*_(*ξ*) are read using the scale markings on the left and the right vertical axes, respectively. The purple and red lines show the wake functions for *β* = 0.1 m and the green and black lines for *β* = 0.9 m, respectively.

Figure 8 shows the approximate wake functions (left) obtained using Eqs (77) and (78) and the exact ones (right) obtained using Eqs (24) and (25) for the shorter bunch case of *σ*_*z*_ = 10 mm. The purple and red lines show the wake functions for *β* = 0.1 m and the green and black lines for *β* = 0.9 m, respectively. For the direct space-charge wake functions *W*_*T*,*direct*_(*ξ*) (purple solid and green solid), the agreement is relatively good for relativistic and non-relativistic beams, but agreement of the indirect wake functions *W*_*T*,*indirect*_(*ξ*) (red dashed and black dashed) is poor, especially for the non-relativistic beam (red dashed). Figure 8 shows that Eq. (76) cannot approximate the exact wake function for a non-relativistic short bunch, even if we slice the beam into infinite segments because of the non-negligible longitudinal leakage of the indirect space-charge force to nearby segments. In other words, the two-dimensional approximation cannot, in principle, be used to accurately determine the space-charge force for a non-relativistic beam with shorter bunch length, even if the bunch is sliced to infinite segments. This result has an important implication for simulations of space-charge force of an electron bunch at low energy such as just after a DC gun.

**Figure 9:**
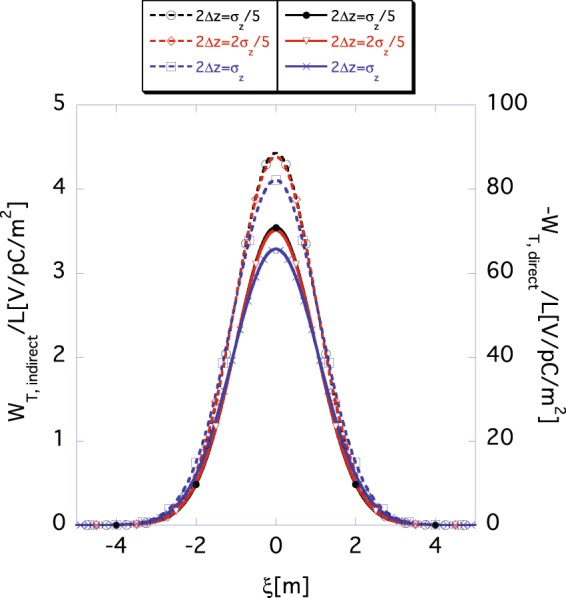
Mesh size 2Δ*z* dependence of indirect wake functions *W*_*T*,*indirect*_(*ξ*) according to Eq. (74) and of direct wake function *W*_*T*,*direct*_(*ξ*) according to Eq. (75) for a beam with *β* = 0.1 and *σ*_*z*_ = 1 m, where *r*_*b*_ = 10 mm and *a* = 40 mm. The dashed (read using the scale markings on the left axis) and the solid (read using the scale markings on the right axis) lines show the indirect *W*_*T*,*indirect*_(*ξ*) and the direct *W*_*T*,*direct*_(*ξ*) space-charge wake functions. The black, red and purple lines show the wake functions for 2Δ*z* = *σ*_*z*_/5, for 2Δ*z* = 2*σ*_*z*_/5, and for 2Δ*z* = *σ*_*z*_, respectively.

It is impractical to slice a bunch into infinite segments. Then, the question is that how finitely must a bunch be sliced to secure good accuracy in case of the two-dimensional approach. To this end, we compare the functions *W*_*T*,*direct*_(*ξ*) and *W*_*T*,*indirect*_(*ξ*) calculated using Eqs (74) and (75) (approximate results) with those calculated using Eqs (24) and (25) (rigorous results) for various segment sizes 2Δ*z*. Let us consider a long bunch. Figure 9 shows the direct space-charge wake function *W*_*T*,*direct*_(*ξ*) (solid) and the indirect one *W*_*T*,*indirect*_(*ξ*) (dashed) for a non-relativistic beam with *β* = 0.1 and *σ*_*z*_ = 1 m. The black, red and purple lines show the functions *W*_*T*,*direct*_(*ξ*) and *W*_*T*,*indirect*_(*ξ*) for 2Δ*z* = *σ*_*z*_/5, for 2Δ*z* = 2*σ*_*z*_/5, and for 2Δ*z* = *σ*_*z*_, respectively. The exact direct wake function *W*_*T*,*direct*_(*ξ*) and indirect one *W*_*T*,*indirect*_(*ξ*) are represented by the purple solid and the red dashed lines in Fig. 7, respectively. When the segment size 2Δ*z* is equal to 2*σ*_*z*_/5 or smaller, the approximate wake functions *W*_*T*,*direct*_(*ξ*) and *W*_*T*,*indirect*_(*ξ*) (black and red lines) reproduce the exact ones, as shown in Fig. 7 with good accuracy. By contrast, when the segment size 2Δ*z* is identical to the rms size *σ*_*z*_ (purple lines), the functions *W*_*T*,*direct*_(*ξ*) and *W*_*T*,*indirect*_(*ξ*) deviate significantly from the exact ones.

**Figure 10:**
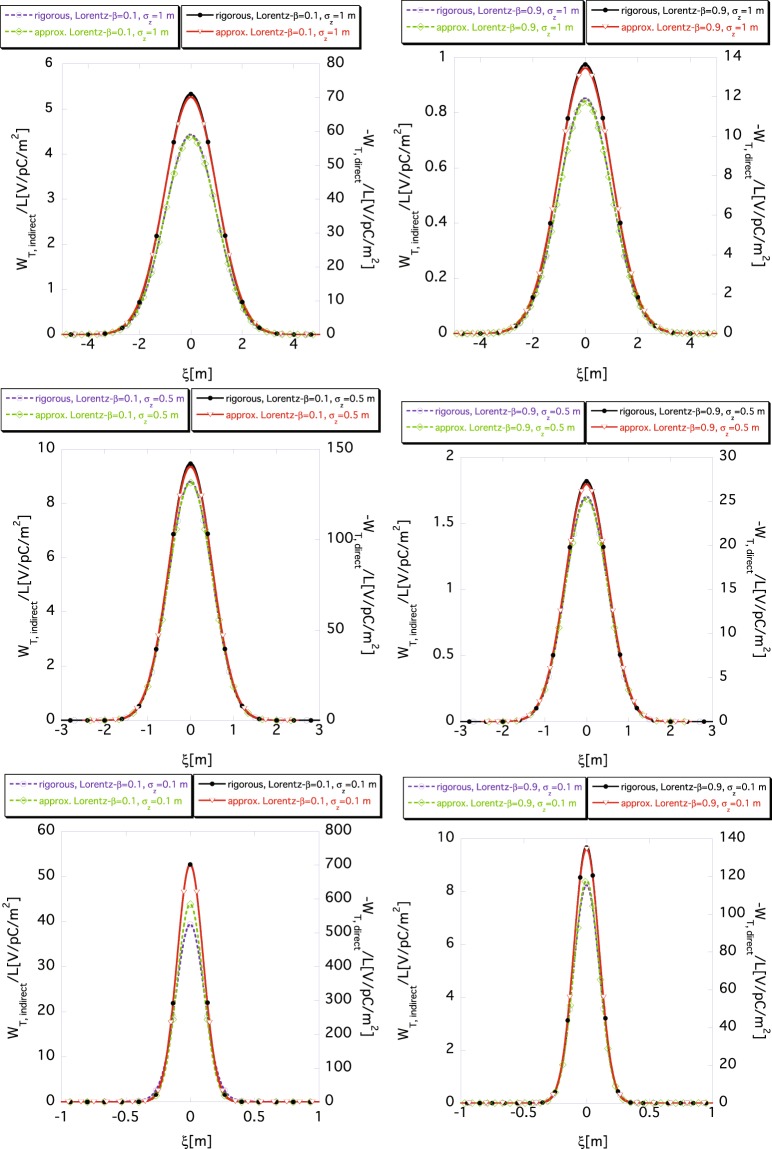
The rms bunch length *σ*_*z*_ dependence of *W*_*T*,*indirect*_(*ξ*) according to Eq. (75) and *W*_*T*,*direct*_(*ξ*) according to Eq. (74) for fixed segment size 2Δ*z* = 2*σ*_*z*_/5, where *r*_*b*_ = 10 mm and *a* = 40 mm. The upper, middle and lower panels show the wake functions for *σ*_*z*_ = 1 m, for *σ*_*z*_ = 0.5 m, and for *σ*_*z*_ = 0.1 m, respectively. The left and right panels show the wake functions for *β* = 0.1, and for *β* = 0.9, respectively. The dashed (read using the scale markings on the left axis) and solid lines (read using the scale markings on the right axis) show the indirect *W*_*T*,*indirect*_(*ξ*) and the direct *W*_*T*,*direct*_(*ξ*) space-charge wake functions, respectively. The black and purple lines show the exact wake functions obtained according to Eqs (24) and (25), respectively. The red and green lines show the approximate wake functions obtained using Eqs (74) and (75), respectively.

Now, let us fix the segment size to 2Δ*z* = 2*σ*_*z*_/5 and compare the approximate wake functions with the exact ones for different bunch lengths. Figure 10 shows the *σ*_*z*_-dependence of *W*_*T*,*indirect*_(*ξ*) according to Eq. (75) and that of *W*_*T*,*direct*_(*ξ*) according to Eq. (74) for different Lorentz-*β*. The upper, middle and lower panels show the wake functions *W*_*T*,*indirect*_(*ξ*) and *W*_*T*,*direct*_(*ξ*) for *σ*_*z*_ = 1 m, for *σ*_*z*_ = 0.5 m, and for *σ*_*z*_ = 0.1 m, respectively. The left and the right panels in the figure show the wake functions *W*_*T*,*indirect*_(*ξ*) and *W*_*T*,*direct*_(*ξ*) for *β* = 0.1, and for *β* = 0.9, respectively. The dashed and the solid lines show the indirect wake function *W*_*T*,*indirect*_(*ξ*) and the direct one *W*_*T*,*direct*_(*ξ*), respectively. The black and purple lines show the exact wake functions *W*_*T*,*direct*_(*ξ*) and *W*_*T*,*indirect*_(*ξ*) determined using Eqs (24) and (25), respectively, while the red and green lines show the approximate ones obtained using Eqs (74) and (75), respectively. In most cases, the approximate wake functions *W*_*T*,*direct*_(*ξ*) and *W*_*T*,*indirect*_(*ξ*) well reproduce the exact ones by selecting the segment size as 2Δ*z* = 2*σ*_*z*_/5. The approximate result of the indirect space-charge wake function *W*_*T*,*indirect*_(*ξ*) with *σ*_*z*_ = 0.1 m and *β* = 0.1 (bottom left), for the non-relativistic and shorter bunch case, shows the most significant deviation from the exact indirect wake functions *W*_*T*,*indirect*_(*ξ*). This deviation is reduced as the beam becomes more relativistic, as shown in the result for the beam with *β* = 0.9 and *σ*_*z*_ = 0.1 m (bottom right).

In this parameter set, if the rms beam size *σ*_*z*_ is larger than 0.5 m, the accuracy of the two-dimensional slicing approach is ensured by setting the segment size to 2Δ*z* = 2*σ*_*z*_/5 or smaller.

Finally, let us examine how the exact formula (23) of the wake function with finite *σ*_*z*_ can be approximated using formula (73). To this end, consider the frequency region relevant to the formulas (23) and (73), which are expressed by Eqs (27) and (80), respectively. For the beam with finite bunch length, formulas (23) and (73) for the wake functions are dominantly specified by the impedances (Eqs (27) and (80)) in this frequency region:

81 $$X\equiv \frac{|\omega |{\sigma }_{z}}{c\beta }\lesssim \sqrt{2}.$$

As demonstrated by the results in Figs 8 and 10, condition (81) means that the exact formula (23) becomes more significant for describing the wake function *W*_*T*_(*ξ*), as the bunch length decreases because the upper limit of the frequency increases.

Equating formula (27) to (80) yields the condition under which the exact wake function (23) can be well approximated by the wake function (73). This condition is expressed as

82 $$\frac{X}{{\sigma }_{z}\gamma {r}_{b}}[{K}_{1}(\frac{X{r}_{b}}{{\sigma }_{z}\gamma })-\frac{{K}_{1}(\frac{Xa}{{\sigma }_{z}\gamma })}{{I}_{1}(\frac{Xa}{{\sigma }_{z}\gamma })}{I}_{1}(\frac{X{r}_{b}}{{\sigma }_{z}\gamma })]\approx \frac{{\sin }^{2}\,\frac{X{\rm{\Delta }}z}{{\sigma }_{z}}}{{(\frac{X{\rm{\Delta }}z}{{\sigma }_{z}})}^{2}}\,{\rm{factor}},$$

83 $${\rm{factor}}\equiv (\frac{1}{{r}_{b}^{2}}-\frac{1}{{a}^{2}}),$$

for $0 < X\lesssim \sqrt{2}$. For a long bunch such as

84 $$\frac{{r}_{b}}{{\sigma }_{z}}\ll 1,$$

85 $$\frac{a}{{\sigma }_{z}}\ll 1,$$

condition (82) can be approximated as

86 $$\frac{{\sin }^{2}\,\frac{X{\rm{\Delta }}z}{{\sigma }_{z}}}{{(\frac{X{\rm{\Delta }}z}{{\sigma }_{z}})}^{2}}\approx 1,$$

which substantially provides the condition for the mesh size relative to the bunch length.

Indeed, as most of the results in Fig. 10 show, when the condition

87 $$2{\rm{\Delta }}z\lesssim \frac{2}{5{\sigma }_{z}},$$

is satisfied, the approximate wake functions given by Eq. (73) well reproduce the exact ones given by Eq. (23) for the long bunch. For a relativistic beam, Eq. (82) reproduces the same condition as that given in (86), as well, which means Eq. (73) well approximates the exact formula (23).

However, for a non-relativistic beam with a short bunch, such as

88 $$\frac{{r}_{b}}{{\sigma }_{z}\gamma }\gtrsim 1,$$

89 $$\frac{a}{{\sigma }_{z}\gamma }\gtrsim 1,$$

the wake function (73) cannot approximate the exact function (23), even if the mesh size is infinitesimal. The results for *a* = 40 mm and *σ*_*z*_ = 0.1 m in Fig. 10 demonstrate that even the condition

90 $$\frac{a}{{\sigma }_{z}\gamma }\sim 0.4,$$

is insufficient for applying Eq. (73) to produce the exact wake functions. Hence, Eq. (73) fails to generate accurate wake functions for a non-relativistic beam with a short bunch, even when condition (87) is satisfied.

## Summary

The space-charge wake function is conventionally described by the *δ*-function, assuming that the imaginary part of the impedance is constant over frequency. This may be a good approximation for an ultra-relativistic beam, but because the space-charge force is more important in the case of a non-relativistic beam, we need more rigorous descriptions of the space-charge wake functions for any magnitude of beam energy.

To this end, we calculated the space-charge wake function directly in the time domain three-dimensionally by assuming a longitudinal Gaussian bunch. In addition, we derived the space-charge impedance by performing Fourier transformation of the wake function. Then, we compared the impedance calculated using the proposed method with that calculated using Gluckstern’s formula for a non-relativistic beam and found some discrepancy. This discrepancy was ascribed to Gluckstern’s use of an approximate definition of the transverse impedance. The proposed impedance formula leads to more rapid damping of the impedance at high frequencies than that achieved with Gluckstern’s formula. As a result, the correct transverse wake function is more deformed relative to the *δ*-function than previously thought based on Gluckstern’s formula.

The space-charge wake function *W*_*T*_(*ξ*) can be divided into the direct *W*_*T*,*direct*_(*ξ*) and the indirect *W*_*T*,*indirect*_(*ξ*) components. The indirect wake function originates from the image charge on the chamber wall that spread out by 2*a*/*γ* where *a* is the chamber radius. As *a* increases, the indirect space-charge wake function decreases and becomes broader.

In typical numerical simulations of the space-charge force, a bunch is sliced into many longitudinal segments and the space-charge force is calculated two-dimensionally for each segment. Because we now have the three-dimensionally correct space-charge wake function, we can examine the accuracy of the two-dimensional approach and determine how finely must a bunch be sliced to secure a good approximation.

We investigated Lorentz-*β* dependence, as well as the longitudinal rms beam size *σ*_*z*_ dependence of the wake function *W*_*T*_(*ξ*). We found that the conventional two-dimensional approach provides a good approximation if the slicing segment size 2Δ*z* is smaller than 2*σ*_*z*_/5. One exceptional case is non-relativistic beams with a short bunch (for example, *β* = 0.1 and *σ*_*z*_ = 0.1 m), where the longitudinal leakage of the space-charge fields to nearby segments cannot be ignored. For accurate simulation of the space-charge force for such a beam, that is, low energy electron beams just after a DC gun, a more rigorous three-dimensional approach is indispensable.

## Electronic supplementary material

Supplementary information
