# Supplementary material for: Rigorous formulation of space-charge wake function and impedance by solving the three-dimensional Poisson equation
Source: Sci Rep. 2018 Aug 24;8:12805. doi: 10.1038/s41598-018-30960-2 (PMC6109189; doi:10.1038/s41598-018-30960-2)
Supplement: Supplementary file 1 — Supplementary information [file 41598_2018_30960_MOESM1_ESM.pdf]

Rigorous formulation of space charge wake function and impedance by solving three-dimensional Poisson equation

Yoshihiro Shobuda[1,*] **and Yong Ho Chin**[2]

[1]JAEA, 2-4 Shirakata, Tokaimura, Nakagun, Ibaraki 319-1195, JAPAN
[2]KEK, High Energy Accelerator Research Organization, 1-1 Oho, Tsukuba, Ibaraki 305-0801, JAPAN
[*]yoshihiro.shobuda@j-parc.jp
[+]these authors contributed equally to this work

ABSTRACT

In this Appendix, we derive two Green functions, which are given by Eqs.(9) and (60) in the text.

A Derivation of Green function

A.1 For three dimensional case

Let us solve

$$\triangle G(\vec{r},\vec{r}') = -\delta(\vec{r}-\vec{r}'), \tag{1}$$

by using a cylindrical coordinate (ρ,θ,z), where $\vec{r} = (\rho,\theta,\bar{z}), \vec{r}' = (\rho',\theta',\bar{z}')$, $\triangle$ and $\delta(\vec{r})$ denote the three-dimensional Laplacian and three-dimensional δ-function, respectively. The Green function $G(\vec{r},\vec{r}')$ must satisfy the boundary condition that $G(\vec{r},\vec{r}')$ is equal to zero at $\rho = a$, where $= a$ is the chamber radius. From here on, we confine our discussion to $\rho < a$.

First, we neglect the boundary condition. For the case at hand, the Green function in the cylindrical coordinate is given by[1,2]

$$G^N(\vec{r},\vec{r}') = \sum_{m=0}^{\infty} \frac{\varepsilon_m}{2\pi^2} \cos[m(\theta-\theta')] \begin{cases} \int_0^\infty d\lambda \cos[\lambda(z-z')] I_m(\lambda\rho) K_m(\lambda\rho'), & \text{for } \rho' > \rho, \\ \int_0^\infty d\lambda \cos[\lambda(z-z')] I_m(\lambda\rho') K_m(\lambda\rho), & \text{for } \rho' < \rho, \end{cases} \tag{2}$$

where $\varepsilon_m = 2-\delta_{m0}$ and δ_{mn} is the Kronecker-δ.

The solution of the homogeneous equation corresponding to Eq.(1), which has no singularity at $\rho = 0$ and satisfies the reciprocal condition[1,2]

$$G(\vec{r},\vec{r}') = G(\vec{r}',\vec{r}), \tag{3}$$

can be expressed as[2]

$$G^H(\vec{r},\vec{r}') = \sum_{m=0}^{\infty} \cos m(\theta-\theta') \int_0^\infty d\lambda F_m(\lambda) I_m(\lambda\rho) I_m(\lambda\rho') \cos\lambda(z-z'), \tag{4}$$

where $F_m(\lambda)$ is an arbitrary function.

General Green functions are obtained by adding Eq.(2) and (4). Then, the Green function satisfying the boundary condition provides $F_m(\lambda)$ as

$$F_m(\lambda) I_m(\lambda a) + \frac{\varepsilon_m}{2\pi^2} K_m(\lambda a) = 0. \tag{5}$$

Finally, the Green function $G(\vec{r},\vec{r}')$ for $\rho < a$ that satisfies the boundary condition $G = 0$ at $\rho = a$ is given by[2]

$$G(\vec{r},\vec{r}') = \sum_{m=0}^{\infty} \frac{\varepsilon_m}{2\pi^2} \cos m(\theta-\theta')$$
$$\times \begin{cases} \int_0^\infty d\lambda \left[K_m(\lambda\rho') - \frac{K_m(\lambda a)}{I_m(\lambda a)} I_m(\lambda\rho')\right] I_m(\lambda\rho)\cos\lambda(\bar{z}-\bar{z}'), & \text{for } \rho' > \rho, \\ \int_0^\infty d\lambda \left[K_m(\lambda\rho) - \frac{K_m(\lambda a)}{I_m(\lambda a)} I_m(\lambda\rho)\right] I_m(\lambda\rho')\cos\lambda(\bar{z}-\bar{z}'), & \text{for } \rho' < \rho, \end{cases} \tag{6}$$

which is identical to Eq.(9) in the text.

A.2 For two dimensional case

Let us solve

$$\triangle^{(2)} G_{two}(\vec{r},\vec{r}') = -\delta^{(2)}(\vec{r}-\vec{r}'), \tag{7}$$

by using a polar coordinate (ρ,θ), where $\vec{r} = (\rho,\theta), \vec{r}' = (\rho',\theta')$, $\triangle^{(2)}$ and $\delta^{(2)}(\vec{r})$ denote the two-dimensional Laplacian and two-dimensional δ-function, respectively, The Green function $G_{two}(\vec{r},\vec{r}')$ must satisfy the boundary condition that $G_{two}(\vec{r},\vec{r}')$ is equal to zero at $\rho = a$. From here on, we confine our discussion to $\rho < a$.

Similarly to the previous case, first, we consider the case neglecting the boundary condition. For this case, the Green function is expressed as[1,2]

$$G^N_{two}(\vec{r},\vec{r}') = \begin{cases} \frac{1}{2\pi}\log(\frac{1}{\rho'}) + \frac{1}{2\pi}\sum_{m=1}^{\infty}\frac{1}{m}(\frac{\rho}{\rho'})^m \cos[m(\theta-\theta')], & \text{for } \rho' > \rho, \\ \frac{1}{2\pi}\log(\frac{1}{\rho}) + \frac{1}{2\pi}\sum_{m=1}^{\infty}\frac{1}{m}(\frac{\rho'}{\rho})^m \cos[m(\theta-\theta')], & \text{for } \rho' < \rho. \end{cases} \tag{8}$$

The solution of the homogeneous equation corresponding to Eq.(7), which has no singularity at $\rho = 0$ and satisfies the reciprocal condition, can be expressed as[2]

$$G^H_{two}(\vec{r},\vec{r}') = \sum_{m=0}^{\infty} \cos[m(\theta-\theta')] c_m \rho^m \rho'^m, \tag{9}$$

where c_m is an arbitrary constant. Because general Green functions are obtained by adding Eqs.(8) and (9), the boundary condition provides c_m as

$$c_m = \begin{cases} -\frac{1}{2\pi}\log(\frac{1}{a}), & \text{for } m = 0, \\ -\frac{1}{2\pi m a^{2m}}, & \text{for } m > 0. \end{cases} \tag{10}$$

Finally, the Green function $G_{two}(\vec{r},\vec{r}')$ for $\rho < a$ in the two dimensional space, which satisfies the boundary condition $G_{two} = 0$ at $\rho = a$ is given by[2]

$$G_{two}(\vec{r},\vec{r}') = \begin{cases} -\frac{1}{2\pi}\log\frac{\rho'}{a} + \frac{1}{2\pi}\sum_{m=1}^{\infty}\frac{\cos m(\theta-\theta')}{m}\rho^m\left(\frac{1}{\rho'^m} - \frac{\rho'^m}{a^{2m}}\right), & \text{for } \rho' > \rho, \\ -\frac{1}{2\pi}\log\frac{\rho}{a} + \frac{1}{2\pi}\sum_{m=1}^{\infty}\frac{\cos m(\theta-\theta')}{m}\rho'^m\left(\frac{1}{\rho^m} - \frac{\rho^m}{a^{2m}}\right), & \text{for } \rho' < \rho, \end{cases} \tag{11}$$

which is identical to Eq.(60) in the text.

References

1. Jackson, J. D. *Class. Electrodyn. 3rd Ed.* **Ch3**, 95–144 (Wiley, New York, 1999).
2. Imamura, T. *Butsuri to Green Kansu* **Ch5**, 75–108 (Iwanami, Tokyo, 1978).
